# The Maternal Microbiome in Pregnancy: From Physiological Changes to Dysbiosis and Obstetrical Complications—Therapeutic Perspectives

**DOI:** 10.3390/life16061033

**Published:** 2026-06-21

**Authors:** Lucia Maria Procopciuc, Gabriela Valentina Caracostea, Adriana Corina Hangan, Roxana Liana Lucaciu

**Affiliations:** 1 Department of Medical Biochemistry, Faculty of Medicine, “Iuliu-Hațieganu” University of Medicine and Pharmacy, 400349 Cluj-Napoca, Romania; lprocopciuc@umfcluj.ro; 2 Medicover Hospital, 407062 Suceagu, Romania; caracostea1@yahoo.com; 3 Department of Inorganic Chemistry, Faculty of Pharmacy, “Iuliu-Hațieganu” University of Medicine and Pharmacy, 400012 Cluj-Napoca, Romania; 4 Department of Pharmaceutical Biochemistry and Clinical Laboratory, Faculty of Pharmacy, “Iuliu-Hațieganu” University of Medicine and Pharmacy, 400349 Cluj-Napoca, Romania; liana.lucaciu@umfcluj.ro

**Keywords:** vaginal microbiota, gut microbiota, oral microbiota, obstetrical complications

## Abstract

During pregnancy, hormonal, metabolic, and immunological changes influence the composition and function of maternal microbial communities. Increasing evidence suggests that the maternal microbiota—particularly in the vaginal, gut, and oral environments—plays a significant role in maintaining pregnancy homeostasis and supporting fetal development. In healthy pregnancies, the vaginal microbiota is typically dominated by *Lactobacillus* species, which help maintain a low vaginal pH and protect against ascending infections. However, disruption of this balance (vaginal dysbiosis) has been associated with obstetrical complications such as intrauterine infection and preterm birth. Similarly, the maternal gut microbiota undergoes trimester-specific changes that contribute to metabolic adaptations required for fetal growth, while alterations in microbial composition have been linked to metabolic disorders including gestational diabetes mellitus and preeclampsia. Changes in oral microbiota and periodontal disease have also been associated with adverse pregnancy outcomes through systemic inflammatory pathways and potential microbial translocation to the placenta. Recent advances in sequencing technologies have improved the understanding of host–microbiome interactions in pregnancy, although the existence of a placental microbiome remains controversial. Overall, maternal microbiota plays an important role in pregnancy physiology, and its dysregulation may contribute to obstetrical complications. Understanding these mechanisms may facilitate the development of microbiome-based diagnostic and therapeutic strategies in maternal–fetal medicine.

## 1. Introduction

The human microbiota represents a complex and dynamic ecosystem composed of trillions of microorganisms, including bacteria, viruses, fungi, and archaea, that colonize multiple anatomical sites of the human body. These microbial communities coexist with the host in a symbiotic relationship and play an essential role in numerous physiological processes, including metabolism, immune system development, and protection against pathogenic microorganisms [1]. Advances in high-throughput sequencing technologies, particularly next-generation sequencing and metagenomic approaches, have significantly expanded our understanding of the human microbiome and its involvement in both health and disease [2]. As a result, the study of host–microbiome interactions has become a rapidly growing field in biomedical research, with increasing attention focused on maternal–fetal health [3,4].

Although the maternal microbiota comprises bacteria, viruses, fungi, and archaea, the present review focuses primarily on the maternal bacteriome, defined as the bacterial component of the microbiota [5,6]. This approach was adopted because most evidence linking maternal microbial communities to pregnancy physiology and obstetrical complications derives from studies based on bacterial profiling using 16S rRNA gene sequencing and metagenomic analyses.

Dysbiosis refers to an alteration in the composition, diversity, stability, or functional activity of microbial communities compared with a healthy physiological state. It may involve depletion of beneficial microorganisms, expansion of opportunistic pathogens, reduced microbial diversity, or disruption of normal host–microbe interactions. During pregnancy, dysbiosis affecting the vaginal, gut, or oral bacteriome has been associated with adverse obstetrical outcomes, including preterm birth, gestational diabetes mellitus, preeclampsia, fetal growth restriction, and intrauterine infection [7,8].

Pregnancy represents a unique physiological state characterized by profound hormonal, metabolic, and immunological adaptations that support fetal growth and maternal tolerance of the semi-allogenic fetus. These changes are necessary to maintain pregnancy while simultaneously protecting both the mother and fetus from infectious threats [9,10]. Increasing evidence suggests that these systemic adaptations are accompanied by dynamic changes in the maternal microbiota across several body sites, including the gastrointestinal tract, vaginal environment, and oral cavity [11,12,13]. These microbial communities are thought to contribute to maternal physiological adaptation to pregnancy and may play an important role in non-communicable diseases, including obesity, diabetes, allergies, and in shaping fetal development and neonatal health [14,15,16].

The vaginal microbiota has been one of the most extensively studied microbial ecosystems in relation to pregnancy outcomes [17]. In healthy reproductive-age women, the vaginal microbiome is typically dominated by species belonging to the genus *Lactobacillus*, which produce lactic acid and other antimicrobial compounds that maintain a low vaginal pH and inhibit the growth of potentially pathogenic microorganisms [18]. During pregnancy, the vaginal microbiota generally becomes less diverse and more stable, with a predominance of *Lactobacillus* species such as *Lactobacillus crispatus*, *Lactobacillus jensenii*, and *Lactobacillus gasseri* [19]. This microbial stability is believed to provide an important protective barrier against ascending infections that could compromise the intrauterine environment and lead to adverse pregnancy outcomes [20]. However, disruption of the normal vaginal microbial balance, commonly referred to as vaginal dysbiosis, has been associated with several obstetrical complications [21]. One of the most well-established associations is between bacterial vaginosis and preterm birth. Bacterial vaginosis is characterized by a reduction in *Lactobacillus* species and an overgrowth of anaerobic bacteria such as *Gardnerella vaginalis*, *Fannyhessea vaginae* (formerly *Atopobium vaginae*), and *Prevotella* species [22]. This microbial imbalance may facilitate the ascent of microorganisms into the upper genital tract, triggering inflammatory responses that contribute to premature rupture of membranes, chorioamnionitis, and preterm labor [23]. Preterm birth remains one of the leading causes of neonatal morbidity and mortality worldwide, highlighting the importance of understanding the role of microbial factors in its pathogenesis [24]. Recent studies further support the association between vaginal microbiota composition and preterm birth risk, emphasizing the importance of microbial community structure and host–microbe interactions in pregnancy outcomes [25,26].

In addition to the vaginal microbiota, the maternal gut microbiome undergoes significant alterations throughout pregnancy. Several studies have demonstrated that the composition of the gut microbiota changes progressively across the three trimesters of pregnancy [27,28]. These changes often include reduced microbial diversity and increased abundance of bacterial taxa associated with metabolic inflammation. Such alterations may contribute to physiological metabolic adaptations during pregnancy, including increased insulin resistance and enhanced energy storage, which help ensure adequate nutrient supply for fetal development [29]. Nevertheless, excessive or abnormal changes in the gut microbiota may contribute to metabolic disorders such as gestational diabetes mellitus (GDM) and obesity-related complications [30]. Recent research has highlighted the potential role of gut microbial metabolites and microbial diversity in predicting the development of gestational diabetes, suggesting that microbiome analysis could become a useful tool for early risk stratification [25,31]. GDM is one of the most common metabolic complications of pregnancy and has been associated with significant alterations in gut microbial composition [32]. Studies have reported differences in microbial diversity and abundance of specific bacterial taxa in pregnant women with GDM compared with healthy pregnancies. These microbial changes may influence glucose metabolism through multiple mechanisms, including modulation of inflammatory pathways, alterations in SCFAs (short-chain fatty acids) production, and effects on intestinal barrier function [33]. Consequently, the maternal gut microbiota has emerged as a potential contributor to metabolic dysregulation during pregnancy and may represent a promising target for preventive interventions.

The oral microbiota has also been implicated in pregnancy outcomes. Periodontal disease, a chronic inflammatory condition characterized by dysbiosis of the oral microbial community, affects a substantial proportion of pregnant women and has been associated with adverse pregnancy outcomes such as preterm birth, low birth weight, and preeclampsia [34]. It has been proposed that periodontal pathogens may enter the systemic circulation and reach the placental environment through hematogenous dissemination, thereby inducing inflammatory responses that affect pregnancy progression [35]. Moreover, maternal oral health may influence systemic inflammatory pathways involved in pregnancy disorders [36].

Another area of ongoing scientific debate concerns the existence of a stable placental microbiome. Early sequencing studies reported the presence of bacterial DNA in placental tissues and suggested that the placenta may harbor a low-abundance microbial community, possibly resembling the oral microbiota [37]. However, subsequent investigations have questioned these findings and emphasized the methodological challenges inherent to low-biomass microbiome research, including contamination from laboratory reagents, environmental sources, sampling procedures, and sequencing workflows [38,39,40]. Several studies have demonstrated that microbial signals detected in placental samples may be difficult to distinguish from background contamination, particularly when bacterial biomass is extremely low. Consequently, although bacterial DNA and microbial components have occasionally been identified in placental tissues, current evidence does not conclusively support the existence of a consistent resident placental microbiome in healthy pregnancies [39,40]. Further studies employing rigorous contamination controls and standardized methodologies are required to clarify this issue. Despite this controversy, microbial invasion of the amniotic cavity and intrauterine infections remain well-recognized contributors to adverse pregnancy outcomes, particularly preterm birth and chorioamnionitis [41]. Ascending infections from the lower genital tract are considered one of the primary mechanisms through which microorganisms may reach the fetal environment [42].

Accumulating evidence suggests that microbial dysbiosis may contribute to obstetrical complications through several biological mechanisms. One of the most important mechanisms involves activation of inflammatory pathways. Microbial components such as lipopolysaccharides and other pathogen-associated molecular patterns can activate maternal immune responses through pattern recognition receptors, leading to the production of pro-inflammatory cytokines [43]. Excessive inflammation during pregnancy has been implicated in the pathogenesis of several complications, including preterm labor, preeclampsia, and fetal growth restriction (FGR) [44,45].

In addition to immune activation, microbial metabolites produced by the gut microbiota may also influence pregnancy outcomes [46]. SCFAs, including acetate, propionate, and butyrate, are produced through bacterial fermentation of dietary fibers and play an important role in regulating metabolic and immune functions [33]. Alterations in the production of these metabolites may contribute to metabolic disturbances, systemic inflammation, and endothelial dysfunction, all of which are relevant to the development of pregnancy complications such as gestational diabetes and hypertensive disorders of pregnancy [47].

Furthermore, disruption of epithelial barrier integrity represents another potential mechanism linking microbiota dysbiosis to adverse pregnancy outcomes. The intestinal barrier normally prevents translocation of microbial components into the systemic circulation. However, alterations in gut microbiota composition may impair barrier function, allowing microbial products to enter the bloodstream and promote systemic inflammation. Such processes may influence maternal metabolic regulation and immune responses during pregnancy [48].

Given the increasing recognition of the maternal microbiome as an important determinant of pregnancy health, understanding the complex interactions between host physiology and microbial communities has become a key area of research in maternal–fetal medicine. Advances in microbiome research may provide valuable insights into the pathogenesis of obstetrical complications and open new perspectives for preventive and therapeutic interventions [49]. Potential strategies include dietary interventions, probiotic and prebiotic supplementation, and personalized microbiome-based approaches.

The aim of this review is to summarize current evidence regarding the role of the maternal microbiota in obstetrical complications. Particular attention is given to microbial alterations in the vaginal, gut, and oral microbiota and their potential contribution to adverse pregnancy outcomes. In addition, the biological mechanisms linking microbial dysbiosis to pregnancy complications and the emerging clinical implications of microbiome research in obstetrics are discussed.

## 2. Literature Search Strategy

This narrative review was based on a comprehensive literature search conducted in PubMed, Scopus, and Web of Science databases. The search included articles published between January 2010 and March 2026. Keywords used individually and in combination included: “maternal microbiome”, “maternal bacteriome”, “pregnancy”, “vaginal microbiota”, “gut microbiota”, “oral microbiota”, “dysbiosis”, “preterm birth”, “preeclampsia”, “gestational diabetes mellitus”, “fetal growth restriction”, and “pregnancy complications”.

Original research articles, systematic reviews, meta-analyses, and relevant experimental studies published in English were considered. Priority was given to recent studies and high-quality evidence addressing physiological microbiome changes during pregnancy, mechanisms linking dysbiosis to obstetrical complications, and potential therapeutic interventions. Additional references were identified through manual screening of the bibliographies of relevant articles.

## 3. Maternal Microbiota in Healthy and Complicated Pregnancy

Before discussing dysbiosis and pregnancy-related complications, it is important to briefly outline the physiological characteristics of the maternal bacteriome. Although the vaginal, gut, and oral microbial communities differ substantially in their bacterial composition, they share several common physiological functions during pregnancy, including protection against pathogen colonization, maintenance of epithelial barrier integrity, modulation of immune responses, and contribution to metabolic homeostasis. In healthy pregnancy, these microbial ecosystems undergo site-specific adaptations in response to hormonal, metabolic, and immunological changes throughout gestation. As this review focuses on the maternal bacteriome, Table 1 summarizes the dominant bacterial communities inhabiting these body sites during healthy pregnancy, together with the principal trimester-specific physiological changes reported across gestation. Detailed site-specific descriptions and their associations with obstetrical complications are presented in the following sections.

### 3.1. Vaginal Microbiota in Pregnancy

The vaginal microbiota undergoes significant physiological modifications during pregnancy, reflecting complex adaptations that support maternal health and fetal development. These microbial changes are influenced by hormonal fluctuations, immune regulation, and metabolic alterations that occur throughout gestation. Accumulating evidence indicates that the maternal vaginal microbiome plays an essential role in maintaining reproductive tract homeostasis, preventing infections, and regulating immune interactions at the maternal–fetal interface [4,47].

#### 3.1.1. Vaginal Microbiota Composition and Hormonal Regulation

The vaginal microbiota undergoes significant physiological modifications during pregnancy, reflecting adaptations driven by hormonal, metabolic, and immunological changes throughout gestation. These alterations are characterized primarily by increased microbial stability, reduced diversity, and enhanced dominance of *Lactobacillus* species, features that distinguish the vaginal bacteriome of healthy pregnant women from that of non-pregnant individuals. In healthy reproductive-age women, the dominant species include *Lactobacillus crispatus*, *Lactobacillus jensenii*, *Lactobacillus gasseri*, and *Lactobacillus iners* [10,18,51].

During pregnancy, several longitudinal microbiome studies have demonstrated that the vaginal microbiota becomes more stable and less diverse, with increased dominance of *Lactobacillus* species compared with the non-pregnant state [19,28,52]. Pregnancy creates physiological conditions that favor the persistence of *Lactobacillus*-dominated microbial communities, particularly during early and mid-gestation. These bacteria contribute to vaginal health through the production of lactic acid, hydrogen peroxide, and antimicrobial peptides, which help maintain an acidic vaginal environment and inhibit the growth of pathogenic microorganisms [28]. Available data obtained using high-throughput sequencing technologies have confirmed that pregnancy promotes enrichment of *Lactobacillus* species and a reduction in overall microbial complexity [45,53].

Hormonal changes during pregnancy play a central role in shaping the vaginal microbiome. Elevated levels of estrogen and progesterone stimulate glycogen accumulation in vaginal epithelial cells. Glycogen deposited in epithelial cells is broken down into simpler carbohydrates (including glucose and maltose), which can then be utilized by Lactobacillus species and other vaginal bacteria [4,54]. These microorganisms ferment carbohydrates and produce lactic acid, which lowers the vaginal pH to approximately 3.5–4.5, creating an environment unfavorable for pathogenic bacteria. Progesterone also influences immune responses within the vaginal mucosa, contributing to immune tolerance toward commensal microorganisms and fetal tissues. The combined effects of estrogen-mediated glycogen deposition and progesterone-mediated immune modulation create a microenvironment that favors *Lactobacillus* dominance and microbial stability during pregnancy [54,55].

The predominance of *Lactobacillus* species is a distinctive feature of the vaginal bacteriome during pregnancy. *Lactobacilli* maintain an acidic vaginal environment through lactic acid production and contribute to microbial community stability by producing antimicrobial compounds and limiting pathogen colonization [4,56]. These characteristics support the persistence of low-diversity, *Lactobacillus*-dominated communities throughout gestation, a pattern consistently associated with healthy pregnancy [28,57]. These protective mechanisms are particularly important because ascending infections from the lower genital tract represent a major risk factor for complications such as preterm birth, intrauterine infection, and premature rupture of membranes [58].

Although the vaginal microbiota remains relatively stable throughout pregnancy, some subtle temporal changes have been observed. Longitudinal studies have demonstrated that the microbial community tends to maintain *Lactobacillus* dominance across all three trimesters, with relatively low variation compared with non-pregnant women [19,52]. Some studies have reported that microbial diversity decreases during pregnancy, reflecting the establishment of a more homogeneous and protective vaginal environment [45,53].

After delivery, however, the vaginal microbiota undergoes substantial restructuring, often transitioning toward a more diverse microbial composition resembling the non-pregnant state [19]. These postpartum changes are associated with hormonal shifts and restoration of the normal reproductive cycle.

#### 3.1.2. Vaginal Microbiota Dysbiosis in Obstetrical Complications

During pregnancy, *Lactobacillus*-dominated microbiota generally becomes more stable and less diverse, which is thought to protect the uterus and fetal membranes from ascending infections [45]. However, disruption of this balance, referred to as vaginal microbiota dysbiosis, is characterized by a reduction in *Lactobacillus* abundance and increased microbial diversity with proliferation of anaerobic bacteria [45,59]. Studies have identified microorganisms such as *Gardnerella vaginalis*, *Prevotella species*, *Fannyhessea vaginae*, and *Sneathia* species as key taxa linked to adverse pregnancy outcomes [60]. In particular, research examining microbial diversity in pregnancy cohorts demonstrated that certain clades of *Gardnerella* were strongly associated with increased risk of preterm birth [14].

Dysbiosis is frequently associated with bacterial vaginosis (BV). Several studies have demonstrated that increased microbial diversity and reduced *Lactobacillus* abundance were associated with inflammatory processes that contribute to cervical remodeling and premature uterine contractions and have been linked to major obstetrical complications such as preterm birth, premature rupture of membranes, intrauterine infection and chorioamnionitis [28,59,61,62,63]. For example, women who experience preterm birth often exhibit a vaginal microbiota characterized by decreased *Lactobacillus* dominance and increased abundance of anaerobic microorganisms [45]. Furthermore, recent research suggests that the composition of the vaginal microbiome may also influence implantation success, pregnancy maintenance, and neonatal microbial colonization [64,65].

An important characteristic of bacterial vaginosis is the formation of polymicrobial vaginal biofilms. Unlike the *Lactobacillus*-dominated vaginal ecosystem observed during healthy pregnancy, bacterial vaginosis is characterized by structured microbial communities adherent to the vaginal epithelium, primarily composed of *Gardnerella vaginalis* together with other anaerobic bacteria, including *Prevotella* spp., *Mobiluncus* spp., and *Fannyhessea vaginae*. These polymicrobial biofilms protect bacteria from host immune defenses and antimicrobial therapies, facilitating microbial persistence, recurrence of bacterial vaginosis, and maintenance of vaginal dysbiosis. Recent evidence suggests that biofilm formation represents a central pathogenic mechanism in bacterial vaginosis and may contribute to adverse reproductive and obstetrical outcomes through sustained inflammation and disruption of the vaginal microbial ecosystem [66,67]. Similar to oral biofilms implicated in periodontal disease, vaginal polymicrobial biofilms promote microbial resilience and chronic inflammatory responses, highlighting the importance of biofilm-mediated dysbiosis across different maternal microbial niches.

A large longitudinal cohort study conducted by McKee et al. investigated vaginal microbiota composition in pregnant women from multiple cohorts using 16S rRNA gene sequencing. The researchers analyzed microbiome samples collected throughout pregnancy and evaluated their relationship with preterm birth outcomes. The study demonstrated that pregnancies resulting in preterm birth were significantly associated with reduced *Lactobacillus* dominance and increased microbial diversity, particularly communities enriched with anaerobic bacteria. The authors concluded that vaginal microbial composition may serve as an early biomarker for identifying women at increased risk of preterm delivery [68].

Another important investigation by Shen et al. examined vaginal microbiota, inflammatory markers, and metabolic profiles in pregnant women with preterm birth. Using sequencing and metabolomic analyses, the researchers found significant differences in vaginal microbial composition between women who delivered preterm and those who delivered at term. Specifically, the preterm group exhibited altered microbial diversity and metabolic changes associated with inflammatory responses. The study suggested that interactions between microbial communities, host metabolism, and immune signaling may contribute to the pathogenesis of preterm birth [69].

Studies investigating the association between vaginal microbiota dysbiosis and obstetrical complications are presented in Table 2. 

#### 3.1.3. Mechanisms Linking Vaginal Dysbiosis to Obstetrical Complications

Multiple biological mechanisms have been proposed to explain how vaginal microbiota dysbiosis contributes to pregnancy complications.

One of the most widely recognized mechanisms linking vaginal dysbiosis to adverse pregnancy outcomes is ascending infection from the lower genital tract to the uterine cavity. In dysbiotic conditions, pathogenic microorganisms may ascend through the cervix and colonize the fetal membranes, amniotic fluid, and placenta, leading to inflammation and activation of labor pathways [74,75,76]. Studies have identified bacterial taxa such as *Ureaplasma urealyticum*, *Mycoplasma hominis*, *Fusobacterium nucleatum*, and *Gardnerella vaginalis* in the amniotic cavity and placental tissues of pregnancies complicated by preterm birth [19,52]. The cervical mucus plug normally serves as a protective barrier against microbial invasion. However, microbial dysbiosis and inflammation can compromise this barrier by altering mucus composition and degrading mucins through bacterial enzymes such as sialidases and proteases, which are produced by bacteria commonly associated with BV. This degradation facilitates microbial penetration into the upper reproductive tract [77].

Another key mechanism involves activation of maternal immune responses and inflammatory pathways. Dysbiotic microbial communities produce pathogen-associated molecular patterns (PAMPs) such as lipopolysaccharides (LPSs) and lipoteichoic acid, which activate pattern recognition receptors including Toll-like receptors (TLRs) expressed on epithelial and immune cells of the reproductive tract. Upon activation, these receptors initiate intracellular signaling pathways that lead to the production and release of pro-inflammatory cytokines and chemokines [78]. TLRs are key elements of the innate immune system and play an essential role in the body’s initial defense against microbial pathogens [79]. Among the members of the TLR family, TLR4 is the most extensively studied. TLR4 recognizes lipopolysaccharide as well as other PAMP and damage-associated molecular patterns (DAMP) ligands, and its level of activation in the uterus has been associated with both term labor and preterm labor (PTL). Experimental studies in mice have shown that deletion of the TLR4 gene delays parturition and significantly reduces neonatal survival [80]. In addition, pharmacological inhibition of TLR4 signaling using the antagonist (+)-naloxone suppresses the inflammatory cascade triggered by *Escherichia coli* and prevents preterm birth (PTB) [81].

Current evidence highlights the importance of TLR4 expression in decidual endothelial cells. Mice lacking endothelial TLR4 are resistant to LPS-induced PTB, suggesting that the action of TLR4 is not limited to immune cells alone [82]. Clinically, specific maternal single-nucleotide polymorphisms in the TLR4 gene have been strongly associated with extreme preterm delivery, defined as birth before 32 weeks of gestation [83].

TLR4, together with its co-receptor CD14, signals through two major pathways: the MyD88 (Myeloid Differentiation Primary Response 88)-dependent and the TRIF (TIR-domain-containing adapter-inducing interferon-β)-dependent pathways, which regulate different inflammatory gene expression programs. The central role of the MyD88 pathway is supported by findings showing that MyD88-deficient mice are completely protected against *E. coli*-induced preterm birth. Other Toll-like receptors, such as TLR2, also contribute to the regulation of labor timing, and fetal polymorphisms in TLR4 and TLR2 further influence the risk of PTB [84].

Activation of TLRs can promote the assembly of inflammasomes, thereby maintaining the release of inflammatory mediators such as interleukin-6 (IL-6), interleukin-1β (IL-1β), tumor necrosis factor-α (TNF-α), interleukine-8 (IL-8) from placental and decidual tissues [42]. These molecules facilitate the recruitment of immune cells and stimulate the production of prostaglandins and matrix metalloproteinases (MMPs), processes that together contribute to uterine contractility, cervical ripening, and membrane rupture, which are key processes involved in the initiation of PTL [59]. Elevated inflammatory markers have been consistently detected in amniotic fluid and cervical secretions of women experiencing PTB associated with intrauterine infection [85].

In addition, the infiltration of macrophages into the amniotic cavity represents a key pathophysiological event in the initiation of spontaneous labor. This process is largely mediated through NF-κB-dependent transcriptional activation of uterine activation related gene networks, as well as the upregulation of pro-inflammatory cytokines such as TNFα, IL-1β, IL-6, and IL-8 [86]. Overall, pattern-recognition receptors and their downstream signaling pathways play a central role in regulating both normal and pathological mechanisms involved in the onset of labor.

Another mechanism involves microbial metabolites produced by dysbiotic communities. Vaginal dysbiosis can also compromise the integrity of epithelial and mucosal barriers in the reproductive tract. *Lactobacillus* species normally support epithelial health by producing lactic acid and maintaining tight junction integrity between epithelial cells. Loss of *Lactobacillus* dominance may reduce these protective effects and allow pathogens to interact directly with epithelial tissues.

Alterations in vaginal metabolic profiles have been linked to inflammatory responses in the cervicovaginal environment. In addition, bacterial metabolites and enzymes produced by dysbiotic microbiota may damage epithelial cells and disrupt mucosal barriers, facilitating microbial invasion and inflammatory responses. For example, bacterial vaginosis-associated organisms produce metabolites such as amines and SCFAs, which can alter local immune responses and epithelial function [69].

Microbial metabolites also play an important role in the pathophysiology of pregnancy complications. Dysbiotic microbiota may produce metabolites that influence immune signaling pathways and systemic inflammation. These metabolic alterations can affect maternal immune tolerance mechanisms that are essential for maintaining pregnancy. In addition, microbial dysbiosis may lead to increased production of inflammatory mediators and oxidative stress, which can impair placental development and vascular function. These mechanisms have been implicated in conditions such as preeclampsia and fetal growth restriction, although further research is needed to fully clarify these pathways.

**Figure 1:**
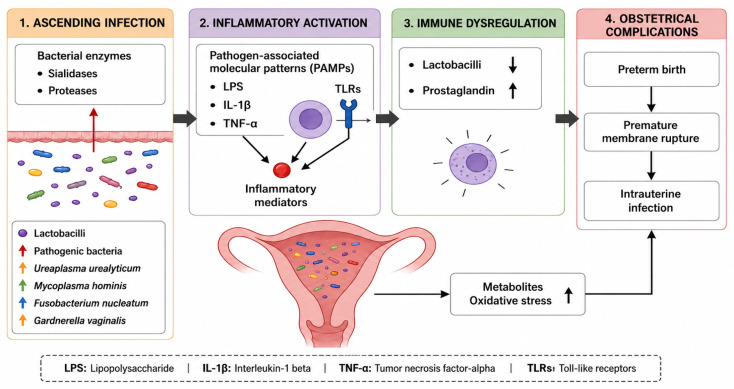
**Mechanisms linking vaginal dysbiosis to pregnancy complications:** Vaginal dysbiosis, characterized by decreased *Lactobacillus* spp. and increased pathogenic bacteria (*Ureaplasma urealyticum*, *Mycoplasma hominis*, *Fusobacterium nucleatum*, and *Gardnerella vaginalis*), may contribute to adverse pregnancy outcomes through several mechanisms. Ascending infection facilitates microbial invasion into the uterine cavity following degradation of cervical mucus by bacterial enzymes such as sialidases and proteases. Dysbiotic microbiota also trigger inflammatory activation through pathogen-associated molecular patterns (PAMPs), including lipopolysaccharides (LPSs), which stimulate Toll-like receptors (TLRs) and induce pro-inflammatory cytokines (IL-1β, TNF-α) and prostaglandin production. In addition, microbial metabolites and increased oxidative stress promote immune dysregulation. Together, these processes may lead to premature rupture of membranes, preterm birth, and intrauterine infection.

Through these mechanisms, including ascending infection, inflammatory activation, epithelial barrier disruption, and immune dysregulation, vaginal dysbiosis may contribute to a range of adverse pregnancy outcomes (Figure 1).

### 3.2. Gut Microbiota in Pregnancy

The maternal gut microbiota undergoes substantial compositional changes during pregnancy. These alterations are characterized by progressive shifts in bacterial diversity and abundance across gestation, particularly during late pregnancy, and are increasingly recognized as important contributors to maternal metabolic adaptation and pregnancy physiology [3,4,13]. Advances in high-throughput sequencing have demonstrated that pregnancy is associated with dynamic shifts in microbial composition, diversity, and metabolic activity across gestation [87].

#### 3.2.1. Trimester-Specific Microbial Shifts

Longitudinal microbiome studies have shown that the composition of the maternal gut microbiota changes progressively during pregnancy. One of the most influential studies conducted by Koren et al. demonstrated that the gut microbiome differs significantly between the first and third trimesters [3]. Early pregnancy is typically characterized by a microbial profile similar to that of non-pregnant women, with a relatively balanced composition dominated by members of the *Firmicutes* and *Bacteroidetes phyla*.

However, during the third trimester, the gut microbiota undergoes a dramatic shift characterized by increased abundance of *Proteobacteria* and *Actinobacteria*, decreased microbial diversity, greater inter-individual variability among pregnant women [3,88].

These changes resemble microbial patterns observed in metabolic syndrome. In pregnancy they appear to represent a physiological adaptation rather than a pathological state. Experimental studies further demonstrated that transplantation of third-trimester gut microbiota into germ-free mice induced increased adiposity and insulin resistance, suggesting that pregnancy-associated microbiota actively contributes to metabolic adaptations [3].

Recent metagenomic studies have confirmed that microbial communities belonging to the families *Lachnospiraceae* and *Ruminococcaceae* undergo substantial changes across pregnancy, particularly during late gestation [45]. These bacteria are involved in the fermentation of dietary fibers and production of SCFAs, which play important roles in host metabolism and immune regulation.

#### 3.2.2. Influence of Gut Microbiota on Maternal Metabolism, Placental Function and Energy Balance

One of the key functions of the maternal gut microbiota during pregnancy is the regulation of energy metabolism. The metabolic demands of pregnancy increase significantly as the fetus grows, requiring adaptations in maternal glucose metabolism, lipid storage, and insulin sensitivity. Gut microorganisms contribute to these adaptations through the production of metabolic compounds such as SCFAs (acetate, propionate, and butyrate) [33].

SCFAs are key metabolites produced by the fermentation of dietary fibers by the maternal gut microbiota. During pregnancy, these microbial metabolites contribute to several physiological adaptations that support maternal metabolism, immune regulation, and fetal development. Increasing evidence suggests that SCFAs play an important role in maintaining metabolic homeostasis and regulating inflammatory responses during gestation [3,4,13]. Also, these microbial metabolites help increase maternal energy storage and support the high metabolic demands of pregnancy [33,89]. Pregnancy is characterized by progressive insulin resistance and increased lipid storage, which help ensure an adequate nutrient supply for the growing fetus.

Experimental studies have shown that SCFAs influence host metabolism through several mechanisms such as stimulation of lipogenesis and fat storage, regulation of glucose homeostasis, modulating insulin signaling pathways, activation of G-protein-coupled receptors (GPR41 and GPR43), which are expressed in adipocytes, intestinal cells, and immune cells and involved in metabolic signaling, modulation of gut hormone secretion, including glucagon-like-peptide-1 (GLP-1) and peptide YY (PYY) [33,88,90,91].

In addition, SCFAs influence the secretion of gut hormones involved in metabolic regulation, including GLP-1 and PYY. These hormones regulate appetite, insulin secretion, and glucose homeostasis, thereby contributing to maternal energy balance during pregnancy [92,93]. Alterations in SCFA production have been associated with metabolic disorders such as GDM and maternal obesity, suggesting that changes in the maternal gut microbiota may influence pregnancy outcomes [25].

Experimental studies and mechanistic investigations suggest that maternal SCFAs may influence fetal immune development and placental function. However, direct evidence demonstrating these effects in human pregnancy remains limited. Microbial metabolites produced in the maternal intestine can reach the placenta and affect placental immune signaling pathways and vascular development. This phenomenon has led to the concept of the gut–placenta axis, which highlights the role of maternal microbiota-derived metabolites in regulating fetal development and pregnancy health [89]. SCFAs also serve as signaling molecules that interact with host receptors and influence gene expression through epigenetic mechanisms.

Taken together, these findings suggest that SCFAs produced by the maternal gut microbiota represent important mediators linking maternal diet, microbial metabolism, immune regulation, and pregnancy outcomes. Understanding the role of these metabolites may provide new insights into the mechanisms underlying metabolic and inflammatory complications during pregnancy.

Recent studies suggest that microbial metabolites contribute to placental nutrient transport, angiogenesis, and immune signaling, thereby influencing fetal growth and development [89]. In addition, microbial-derived folate and other B vitamins participate in the epigenetic regulation of fetal gene expression through one-carbon metabolism and DNA methylation pathways. These mechanisms have been proposed as potential contributors to fetal programming and long-term health outcomes in offspring, although most supporting evidence derives from experimental and preclinical studies [94]. Alterations in microbial metabolic activity have been associated with pregnancy complications such as gestational diabetes mellitus, preeclampsia, and fetal growth restriction [25].

#### 3.2.3. Immune Regulation and Maternal–Fetal Tolerance

In addition to its metabolic functions, the maternal gut microbiota influences immune signaling pathways through microbial metabolites such as SCFAs and tryptophan-derived compounds. These metabolites have been implicated in the regulation of maternal immune adaptation during pregnancy [95]. SCFAs promote the expansion of regulatory T cells (Tregs) and suppress excessive inflammatory responses that could compromise placental development. Butyrate, in particular, acts as a histone deacetylase inhibitor, influencing gene expression and promoting anti-inflammatory immune pathways [33]. Through these mechanisms, microbial metabolites contribute to maintaining immune balance during pregnancy.

Furthermore, bacterial components such as LPSs and other microbe-associated molecular patterns interact with TLRs on immune cells, shaping maternal immune responses throughout pregnancy. Dysregulation of these pathways may contribute to pregnancy complications such as preeclampsia, GDM and preterm birth [96].

Evidence from experimental animal models suggests that maternal gut microbiota-derived metabolites may influence fetal immune development. However, confirmation of these mechanisms in humans is still limited. Bacterial metabolites produced in the maternal intestine can cross the placental barrier and modulate fetal immune cell development, thereby affecting neonatal immune programming [97].

#### 3.2.4. Microbial Synthesis of Essential Vitamins and Bioactive Compounds in Pregnancy

Microbial metabolism in the maternal gut plays a critical role in the synthesis of essential vitamins and bioactive compounds that support maternal health and fetal development. Recent research has also suggested that maternal gut microbiota influences placental function and fetal epigenetic programming, linking maternal nutrition, microbial metabolism, and fetal growth [98]. The intestinal microbiota participates in multiple metabolic pathways that produce micronutrients, cofactors, and signaling molecules involved in cellular metabolism, immune regulation, and fetal growth [33,99,100].

Many intestinal bacteria are capable of synthesizing essential vitamins, particularly B-complex vitamins and vitamin K, which are important for host metabolism and cellular function. Members of the genera *Bacteroides*, *Bifidobacterium*, *Lactobacillus*, and *Enterococcus* contribute to the production of vitamins such as folate (vitamin B9), biotin (vitamin B7), riboflavin (vitamin B2), and cobalamin (vitamin B12) [100,101].

These vitamins act as cofactors in numerous metabolic pathways, including DNA synthesis, amino acid metabolism, and energy production. For example, folate produced by gut bacteria plays an essential role in nucleotide biosynthesis and methylation reactions, processes that are particularly important during pregnancy due to the rapid cell division and tissue growth occurring in the developing fetus [102]. Adequate folate levels are crucial for preventing neural tube defects and supporting placental development. Vitamin K synthesized by intestinal bacteria is also important for blood coagulation and bone metabolism. Microbial production of vitamin K contributes to maintaining maternal coagulation balance and may influence fetal skeletal development [101].

Beyond vitamin synthesis, gut microorganisms produce numerous bioactive metabolites that influence host physiology. These metabolites include along SCFAs, secondary bile acids, amino acid derivatives, and neurotransmitter precursors [33].

Microbial metabolism also contributes to the production of tryptophan-derived metabolites, which activate the aryl hydrocarbon receptor (AhR) pathway and influence immune regulation. Activation of these pathways promotes the development of regulatory immune responses that are essential for maintaining maternal–fetal tolerance during pregnancy [103].

#### 3.2.5. Mechanisms of Placental Transfer

An emerging concept in microbiome research is the maternal–fetal metabolic axis, which describes the bidirectional communication between maternal microbial metabolism and fetal development through the transfer of microbial metabolites across the placenta. Increasing evidence, derived primarily from experimental and animal studies, suggests that metabolites produced by the maternal gut microbiota can enter the maternal circulation, reach the placenta, and potentially influence fetal physiological processes, including immune maturation, metabolic programming, and neurodevelopment [104].

The placenta acts as a selective barrier between the maternal and fetal compartments, but several microbial metabolites produced in the maternal intestine can cross this barrier. Among the most studied are SCFAs, tryptophan-derived indole metabolites, bile acid derivatives, and other microbial signaling molecules [95,105]. These compounds can enter the maternal bloodstream after intestinal absorption and reach the placenta through systemic circulation. Experimental studies have demonstrated that SCFAs generated by microbial fermentation of dietary fibers can cross the placental barrier and reach the fetal circulation. Most evidence regarding placental transfer of microbiota-derived metabolites originates from animal models and experimental studies, whereas direct human data remain relatively scarce. Once transferred to the fetus, these metabolites may serve as energy substrates and signaling molecules that regulate fetal metabolic pathways and immune responses. Experimental studies suggest that SCFAs can interact with fetal immune cells by activating G-protein-coupled receptors and epigenetic pathways, thereby influencing immune cell differentiation and regulatory immune responses [95,106].

In addition to their metabolic effects, microbial metabolites transferred across the placenta may contribute to fetal immune development. Several studies suggest that maternal microbiota-derived metabolites influence fetal immune cell maturation and neonatal immune programming, although the underlying mechanisms remain incompletely understood [107]. Recent research by Morozan et al. demonstrated that maternal microbiota-derived metabolites such as SCFAs and indole derivatives may cross the placental barrier and modulate fetal immune signaling pathways, including NF-κB suppression and Nrf2 activation, thereby promoting anti-inflammatory responses and protecting fetal tissues from oxidative stress [95]. These metabolites have been implicated in microglial maturation and neuroimmune development in experimental models, although their relevance in human pregnancy has not yet been fully established.

Microbial metabolites do not only affect fetal immune development but also influence placental physiology and nutrient transport. Recent experimental studies have shown that microbial-derived vitamins and metabolites can regulate placental angiogenesis and metabolic activity. For example, maternal gut microbiota-derived thiamine has been shown to activate Notch signaling pathways in the placenta, promoting angiogenesis and improving nutrient transport to the fetus [108]. In addition, microbial metabolites can influence placental inflammatory signaling pathways. SCFAs and indole derivatives have been shown to suppress placental inflammation by inhibiting NF-κB-mediated cytokine production, thereby protecting placental tissue from inflammatory damage and supporting normal fetal development [105].

#### 3.2.6. Role of Maternal Gut Microbiota on Fetal Metabolic Programming

Another important consequence of maternal microbial metabolite transfer is its role in fetal metabolic programming. Exposure to microbial metabolites during gestation may influence fetal gene expression that occur without altering the underlying DNA sequence and epigenetic regulation, thereby affecting long-term metabolic and immune health in offspring [102]. These modifications include DNA methylation, histone modification, and regulation by non-coding RNAs [109].

For example, SCFAs can influence histone acetylation and DNA methylation, which regulate gene expression patterns involved in metabolism and immune responses. Microbial metabolites such as butyrate act as histone deacetylase (HDAC) inhibitors, which influence chromatin structure and gene transcription. By modulating histone acetylation, these metabolites can regulate the expression of genes involved in metabolic regulation, immune responses, and energy homeostasis [110,111]. In addition, microbial metabolites can affect DNA methylation pathways by influencing one-carbon metabolism and the availability of methyl donors such as folate and vitamin B12. These processes are essential for normal fetal development and long-term metabolic regulation.

Through these epigenetic mechanisms, maternal gut microbiota has been proposed to influence fetal metabolic pathways and later disease susceptibility. However, much of the available evidence derives from animal studies, and causal relationships in humans remain to be established [25].

**Figure 2:**
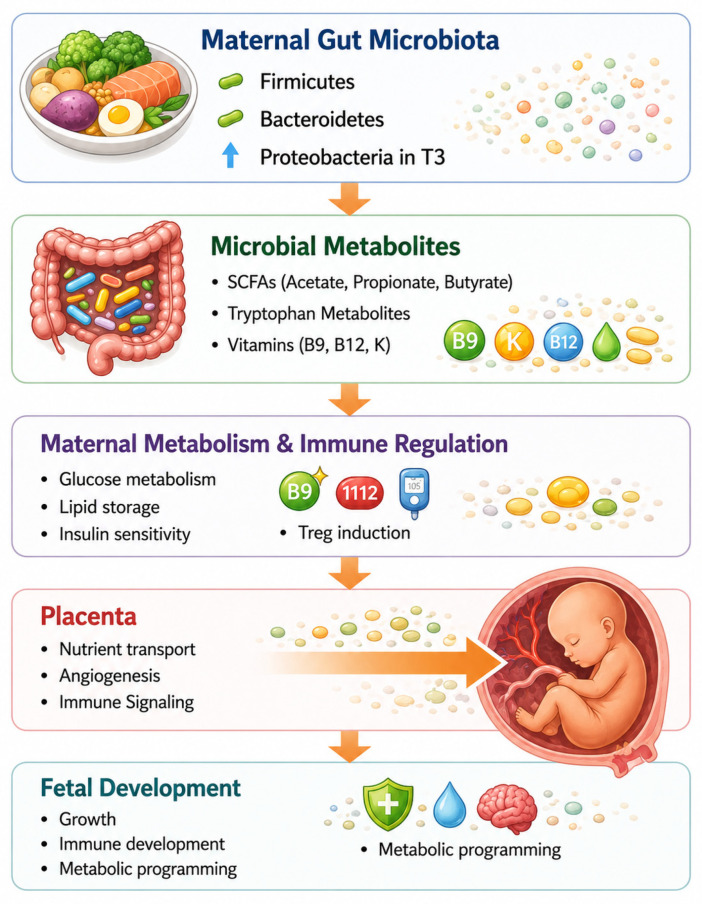
**Conceptual overview of the interactions between maternal gut microbiota and pregnancy physiology.** The maternal gut microbiota, dominated by members of the *Firmicutes* and *Bacteroidetes* phyla with increased *Proteobacteria* during late pregnancy, produces microbial metabolites including short-chain fatty acids (SCFAs: acetate, propionate, and butyrate), tryptophan-derived metabolites, and vitamins (B9, B12, and K). These metabolites influence maternal metabolism and immune regulation by modulating glucose metabolism, lipid storage, insulin sensitivity, and regulatory T-cell responses (Tregs). Microbial metabolites can also reach the placenta through systemic circulation, where they regulate nutrient transport, angiogenesis, and immune signaling. Through these mechanisms, maternal gut microbiota-derived metabolites are proposed to contribute to fetal growth, immune development, and metabolic programming, although several of these pathways are supported predominantly by experimental and preclinical evidence.

Role of the maternal gut microbiota on pregnancy physiology is presented in Figure 2.

#### 3.2.7. Gut Microbiota Dysbiosis and Major Obstetrical Complications

Physiological adaptations occurring during gestation, including hormonal, metabolic, and immunological changes, also influence the composition of the maternal intestinal microbiome. In healthy pregnancies, the gut microbiota is typically dominated by bacterial phyla such as Firmicutes, Bacteroidetes, Actinobacteria, and Proteobacteria, which contribute to immune regulation and metabolic balance [88].

Longitudinal studies have demonstrated that pregnancy is associated with progressive changes in gut microbiota composition, particularly during the third trimester. An increased abundance of Proteobacteria and Actinobacteria and reduced microbial diversity in late pregnancy was observed. Also, maternal gut microbiota may influence metabolic adaptations during gestation [3].

Although many microbial changes observed during pregnancy are considered physiological adaptations, disturbances in the maternal gut microbiota—known as gut dysbiosis—have been associated with several pregnancy complications. Alterations in microbial composition may contribute to metabolic and inflammatory conditions such as GDM, preeclampsia, FGR, maternal obesity and PTB [98]. Dysbiosis may affect pregnancy through multiple mechanisms, including systemic inflammation, increased intestinal permeability, altered microbial metabolite production, and immune dysregulation that may impair placental function and fetal development [3].

Preeclampsia is one of the most extensively studied pregnancy complications associated with maternal gut microbiota dysbiosis. Recent studies have shown that gut microbiota dysbiosis can alter host metabolism and contribute to placental immune dysfunction and impaired vascular remodeling, mechanisms that are implicated in the pathogenesis of preeclampsia [112].

There are studies which investigated risk factors for preeclampsia [113]. Several studies have reported significant alterations in gut microbial composition in women diagnosed with hypertensive disorders of pregnancy. A study conducted by Wang et al. investigated fecal microbiota composition in pregnant women with preeclampsia using 16S rRNA gene sequencing. The researchers compared fecal samples from women diagnosed with preeclampsia with those from healthy pregnant controls. The results demonstrated reduced microbial diversity and increased abundance of *Proteobacteria* and *Bacteroidetes*, along with decreased levels of beneficial bacteria such as *Faecalibacterium* and *Bifidobacterium*. These microbial changes were correlated with elevated inflammatory markers and metabolic disturbances, suggesting that gut microbiota dysbiosis may contribute to the pathogenesis of preeclampsia through inflammatory and immune mechanisms [29].

In a recent animal study, investigators induced gut microbiota dysbiosis in pregnant mice through antibiotic exposure and dietary manipulation. The dysbiotic mice exhibited impaired placental vascularization, increased fetal resorption rates, and abnormal placental development, demonstrating that maternal microbial imbalance can directly affect placental physiology and fetal viability [112].

GDM represents another pregnancy complication strongly associated with gut microbiota alterations. Dysbiosis of intestinal microbial communities may influence maternal glucose metabolism, insulin resistance, and inflammatory responses.

A clinical study by Crusell et al. analyzed fecal samples from pregnant women diagnosed with GDM and healthy pregnant controls using microbiome sequencing techniques. The results demonstrated significant alterations in gut microbiota composition in GDM patients, including reduced microbial diversity and increased abundance of opportunistic bacteria such as *Ruminococcus* and *Blautia*. These microbial changes were associated with metabolic parameters related to insulin resistance [25].

Similarly, Wu et al. performed metagenomic sequencing of fecal samples from pregnant women during the second trimester and identified specific microbial signatures associated with GDM development. The study reported increased abundance of *Firmicutes* and decreased levels of *Bacteroidetes*, suggesting that microbial metabolic pathways involved in carbohydrate metabolism may influence glucose regulation during pregnancy [114]. Although these studies consistently report associations between gut microbiota alterations and GDM, their observational design does not allow causal relationships to be established.

FGR has also been linked to maternal gut microbiota alterations. Several studies suggest that microbial imbalance may affect placental nutrient transport and fetal metabolic programming.

A study investigating maternal gut microbiota composition in pregnancies complicated by FGR identified significant differences in microbial diversity compared with healthy pregnancies. The researchers reported reduced abundance of bacteria involved in SCFAs production, including *Faecalibacterium* and *Roseburia*, which are known to play a role in immune regulation and metabolic homeostasis [115]. SCFAs such as butyrate, propionate, and acetate are important microbial metabolites that influence host metabolism and immune function. Reduced SCFAs production may impair placental nutrient transport and contribute to restricted fetal growth.

PTB has also been associated with alterations in maternal gut microbiota composition. Several studies suggest that gut microbial dysbiosis may contribute to systemic inflammation that triggers premature activation of labor pathways.

A prospective cohort study examining maternal microbiome dynamics during pregnancy demonstrated that shifts in both gut and vaginal microbiota were associated with adverse pregnancy outcomes, including PTB [116]. Women who delivered preterm exhibited increased microbial diversity and enrichment of pro-inflammatory microbial taxa.

Another investigation reported that gut microbiota dysbiosis can promote systemic inflammation through increased production of endotoxins and inflammatory metabolites. These molecules can activate immune signaling pathways that influence uterine immune responses and contribute to premature uterine contractions and cervical remodeling [104].

Collectively, available studies indicate that gut microbiota dysbiosis is consistently associated with metabolic and inflammatory disturbances during pregnancy. Although specific microbial signatures vary across cohorts and analytical approaches, reduced microbial diversity, altered abundance of SCFA-producing bacteria, and enrichment of pro-inflammatory taxa represent the most frequently reported findings in women with GDM, preeclampsia, fetal growth restriction, and PTB. However, most available human data are derived from observational studies, whereas many mechanistic insights originate from experimental and animal models. Therefore, although these associations are biologically plausible, causal relationships remain incompletely established.

Research studies on the role of gut microbiota alterations in pregnancy complications are presented in Table 3.

### 3.3. Oral Microbiota in Pregnancy

The oral cavity harbors one of the most diverse microbial ecosystems in the human body, comprising more than 700 bacterial species, along with fungi, viruses, and protozoa. These microorganisms form complex biofilms on oral surfaces, particularly in dental plaque and gingival tissues [123]. During pregnancy, profound hormonal, immunological, and metabolic changes can alter the composition and behavior of oral microbial communities. These physiological adaptations modify the oral ecological environment, affecting bacterial colonization, biofilm formation, and microbial community composition. As a result, pregnancy is frequently associated with changes in oral microbiota composition and increased susceptibility to inflammatory oral diseases such as pregnancy gingivitis and periodontitis [123,124]. These alterations may influence maternal oral health and have been associated with adverse pregnancy outcomes such as PTB, low birth weight, preeclampsia, and GDM [34,125,126].

#### 3.3.1. Hormonal and Immune Effects on Oral Microbial Communities

Pregnancy induces significant physiological changes that affect the oral environment. One of the primary mechanisms involves elevated levels of estrogen and progesterone, which increase substantially during pregnancy. These hormones affect gingival tissues by increasing vascular permeability and blood flow, leading to gingival edema and increased gingival crevicular fluid. This fluid contains proteins and nutrients that support bacterial growth within dental plaque biofilms [126]. These hormonal fluctuations increase gingival crevicular fluid and promote changes in the oral microbiota composition.

In addition, certain oral microorganisms can directly utilize steroid hormones as growth substrates. Several studies have demonstrated that pregnancy is associated with increased colonization by periodontal pathogens, including: *Porphyromonas gingivalis*, *Prevotella intermedia*, *Fusobacterium nucleatum*, *Treponema denticola*. These microorganisms are key members of the “red complex” periodontal pathogens that contribute to periodontal disease [127].

For example, the periodontal pathogen *Prevotella intermedia* has been shown to use pregnancy-associated steroid hormones as growth factors, leading to increased abundance during pregnancy [128]. Consequently, hormonal changes during pregnancy can favor the proliferation of specific periodontal pathogens and alter the structure of oral microbial communities.

Similarly, *Fusobacterium nucleatum*, a bacterium frequently found in dental plaque, has been detected in placental tissues and has been implicated in intrauterine infections [74].

Longitudinal studies using high-throughput sequencing techniques have shown that pregnancy is associated with increased microbial diversity and shifts toward pro-inflammatory bacterial species in the oral cavity [129]. Hormonal fluctuations also affect the structural properties of gingival tissues. Increased estrogen levels stimulate collagen turnover and connective tissue remodeling, while progesterone influences inflammatory signaling pathways and vascular responses [126].

These physiological changes may weaken epithelial barriers and increase gingival tissue susceptibility to bacterial colonization and inflammation. Consequently, even small amounts of dental plaque may induce significant inflammatory responses in gingival tissues during pregnancy, contributing to the high prevalence of pregnancy gingivitis, a condition affecting up to 60–75% of pregnant women [126]. Pregnancy-associated immunological adaptations may also influence the oral ecosystem by modifying local host–microbe interactions and favoring the expansion of opportunistic periodontal bacteria. These changes occur alongside hormonal alterations and contribute to pregnancy-associated shifts in oral microbial composition [9].

Pregnancy can also influence the composition and properties of saliva, which plays an important role in regulating oral microbial communities. Changes in salivary flow, pH, buffering capacity, and antimicrobial components such as immunoglobulin A (IgA) may influence bacterial adhesion and biofilm formation [130].

Furthermore, metabolic and behavioral factors associated with pregnancy, including increased carbohydrate consumption and pregnancy-related nausea or vomiting, may contribute to changes in the oral environment that favor bacterial growth.

The combined effects of hormonal, immune, and metabolic changes during pregnancy can lead to shifts in oral microbial composition, including increased abundance of periodontal pathogens such as *Porphyromonas gingivalis*, *Prevotella intermedia*, and *Fusobacterium nucleatum* [74,125]. These changes may increase susceptibility to periodontal inflammation and favor the expansion of periodontal-associated bacterial taxa during pregnancy.

#### 3.3.2. Oral-Placental Axis: Translocation of Oral Pathogens to the Placenta. Effects on Placental Function

Periodontal disease is a chronic inflammatory condition characterized by the destruction of the supporting structures of the teeth due to bacterial infection and host immune responses. Several biological mechanisms have been proposed to explain the association between periodontal disease and adverse pregnancy outcomes, such as systemic dissemination of periodontal pathogens, interaction with placental immune receptors and induction of systemic and placental inflammation. Increasing evidence suggests that the oral microbiota may influence pregnancy outcomes through a mechanism known as the oral–placental axis, in which periodontal pathogens originating from the oral cavity disseminate to the placenta and intrauterine environment. This process may trigger inflammatory responses that contribute to pregnancy complications such as PTB, FGR, and preeclampsia [124,125].

One proposed mechanism involves the hematogenous spread of oral bacteria from periodontal lesions to the placenta and amniotic cavity. Periodontal pathogens can enter the bloodstream during routine activities such as chewing or tooth brushing, particularly in individuals with inflamed gingival tissues [131]. Once in the bloodstream, oral pathogens can reach distant organs, including the placenta and stimulate inflammatory responses that can trigger premature labor or impair placental function [34].

Experimental studies have detected oral pathogens such as *Fusobacterium nucleatum*, *Porphyromonas gingivalis*, and *Prevotella species* in placental tissues, amniotic fluid, and fetal membranes, supporting the hypothesis that oral bacteria can reach the intrauterine environment. However, the detection of bacterial DNA or microbial components in placental tissues should not be interpreted as definitive evidence for the existence of a resident placental microbiome. Recent studies have highlighted the methodological challenges associated with low-biomass microbiome analyses and emphasized the importance of rigorous contamination controls. The presence of these bacteria in intrauterine compartments suggests that oral microbes may translocate hematogenously and colonize placental tissues.

In animal models, intravenous inoculation with *Fusobacterium nucleatum* resulted in placental colonization and adverse pregnancy outcomes, including fetal death and preterm delivery [37,74].

Periodontal pathogens may also directly affect placental function by activating immune pathways in placental tissues. After reaching the placenta, bacterial components, such as *Porphyromonas gingivalis*, can interact with immune receptors expressed on trophoblast cells. One of the key mechanisms involves activation of TLRs, particularly TLR2 and TLR4, which recognize bacterial components such as LPSs. Activation of these receptors triggers inflammatory signaling pathways that may impair placental function and fetal growth [132]. These inflammatory processes may disrupt placental vascular development and nutrient transport, potentially contributing to FGR and preeclampsia [133].

Activation of these receptors triggers intracellular signaling pathways that lead to the production of pro-inflammatory cytokines, including: IL-1β, IL-6, TNF-α. Periodontal disease is associated with elevated levels of these pro-inflammatory cytokines. These inflammatory mediators can enter the systemic circulation and influence placental tissues [134]. They can disrupt placental homeostasis, impair trophoblast invasion, and alter placental vascular development [133].

Periodontal pathogens and their endotoxins can also induce systemic inflammatory responses that affect the maternal–fetal interface. Increased circulating levels of inflammatory mediators such as prostaglandin E2 (PGE2), C-reactive protein (CRP), and cytokines have been observed in pregnant women with periodontal disease [134]. These inflammatory molecules can stimulate uterine contractions and cervical ripening, mechanisms that are strongly associated with PTL [42,134]. In addition, chronic inflammation may lead to endothelial dysfunction and oxidative stress, which are key mechanisms involved in the pathogenesis of preeclampsia [135].

Beyond inflammation, oral pathogens may directly impair placental function. Studies have shown that bacterial invasion of placental tissues can disrupt placental angiogenesis, nutrient transport, and immune regulation [133]. For example, infection with *Porphyromonas gingivalis* has been associated with reduced trophoblast invasion and impaired spiral artery remodeling, processes essential for normal placental development. These abnormalities may lead to placental insufficiency and FGR [136].

Furthermore, microbial invasion of the placenta may activate complement pathways and oxidative stress responses that contribute to placental damage and endothelial dysfunction. Additionally, oral pathogens have been detected in placental tissues, amniotic fluid, and umbilical cord blood, further supporting the hypothesis that oral bacteria may contribute to intrauterine infections and pregnancy complications [35].

**Figure 3:**
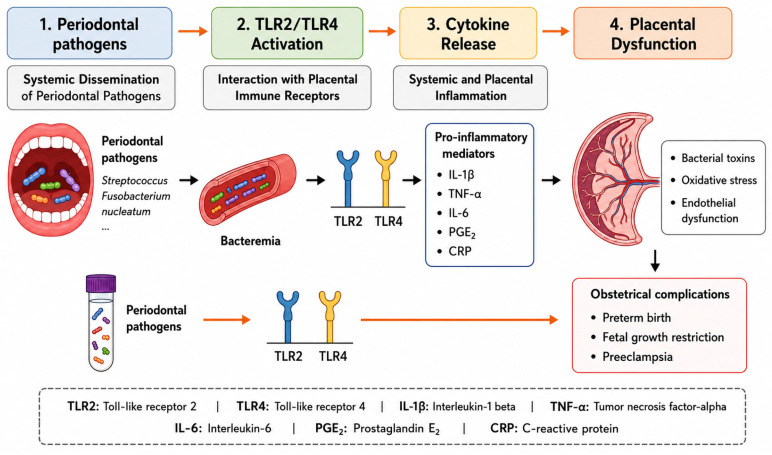
**Mechanisms linking periodontal disease to adverse pregnancy outcomes**. Periodontal pathogens from oral infections may enter the bloodstream and reach the placenta, where they interact with placental immune receptors such as Toll-like receptors (TLR2 and TLR4). This interaction triggers systemic and placental inflammatory responses characterized by increased levels of pro-inflammatory mediators (e.g., IL-1β, TNF-α, IL-6, PGE2, and CRP). These processes can disrupt placental function and contribute to adverse pregnancy outcomes, including preterm birth (PTB), fetal growth restriction (FGR), and preeclampsia.

Mechanisms linking oral dysbiosis to pregnancy complications is presented in Figure 3.

### 3.4. Oral Microbiota Dysbiosis and Major Obstetrical Complications

Under physiological conditions, the oral microbiota maintains a balanced microbial community that contributes to oral and systemic health. However, disturbances in this microbial balance, oral microbiota dysbiosis, may lead to periodontal diseases and systemic inflammatory responses that can influence pregnancy outcomes. Increasing evidence suggests that oral dysbiosis during pregnancy is associated with several obstetrical complications, including PTB, preeclampsia, low birth weight, and intrauterine infections [137,138].

One of the most widely studied conditions linking oral dysbiosis to pregnancy complications is periodontal disease, a chronic inflammatory condition caused by pathogenic bacterial biofilms in periodontal tissues. As discussed in the oral–placental axis section, periodontal pathogens may influence pregnancy outcomes through hematogenous dissemination and activation of inflammatory pathways. Consequently, increasing evidence suggests that maternal periodontal disease is associated with several adverse obstetrical outcomes [34].

Several observational and clinical studies have reported an association between maternal periodontal disease and an increased risk of preterm birth. Epidemiological studies indicate that periodontal pathogens and their inflammatory products may contribute to premature activation of labor pathways. One important study demonstrated that oral pathogens such as *Fusobacterium nucleatum* can translocate from the oral cavity to the placenta through hematogenous dissemination. Han et al. identified *Fusobacterium nucleatum* in placental tissues of women with PTB, suggesting that oral bacteria may reach the intrauterine environment and trigger inflammatory responses leading to PTL [35]. More recent microbiome-based studies have also identified specific oral bacterial genera associated with PTB. Vidmar Šimic et al. analyzed oral microbiota samples from pregnant women using 16S rRNA sequencing and reported that increased abundance of *Prevotella*, *Veillonella*, and *Capnocytophaga* was associated with spontaneous preterm delivery [139]. Another study analyzing salivary microbiota in pregnant women demonstrated that women who delivered preterm or had low birth weight infants exhibited greater instability and heterogeneity in their oral microbiome, suggesting that oral microbial imbalance may contribute to pregnancy complications through inflammatory mechanisms [140].

Although causality remains under investigation, the detection of oral pathogens in placental tissues and the mechanistic evidence from experimental models strongly support the concept that oral microbiota may influence pregnancy outcomes through the oral–placental axis.

Preeclampsia, a pregnancy complication characterized by hypertension and endothelial dysfunction, is another obstetrical complication that has been linked to maternal oral microbiota dysbiosis. Studies have shown that periodontal pathogens and inflammatory mediators may contribute to systemic endothelial activation and oxidative stress, mechanisms involved in the pathogenesis of preeclampsia [135].

Several studies have reported a higher prevalence of periodontal disease among women diagnosed with preeclampsia compared with healthy pregnant controls. Research conducted by Gare et al. reported that severe periodontal disease was significantly associated with increased risk of preeclampsia. Periodontal pathogens such as *Porphyromonas gingivalis*, *Campylobacter rectus*, and *Filifactor alocis* have been proposed as potential contributors to systemic inflammatory responses and endothelial dysfunction, which are considered important mechanisms in the pathophysiology of preeclampsia [141]. In addition, studies examining microbial signatures in oral samples from pregnant women have identified bacterial taxa associated with hypertensive disorders of pregnancy. These microorganisms produce virulence factors and inflammatory mediators that may contribute to placental inflammation and vascular dysfunction [142].

Low birth weight has also been associated with alterations in maternal oral microbiota composition. A clinical study investigating oral microbial profiles in pregnant women analyzed oral microbiome samples using sequencing technologies and identified microbial patterns associated with low birth weight deliveries. The researchers reported that decreased abundance of commensal oral bacteria such as *Neisseria* and increased abundance of inflammatory bacteria were associated with higher risk of delivering low birth weight infants. These findings suggest that oral microbial imbalance may influence fetal growth through inflammatory and metabolic pathways [143].

Emerging evidence suggests that periodontal disease may also be associated with GDM. Chronic inflammation induced by periodontal infection may contribute to insulin resistance and metabolic dysregulation during pregnancy [144]. Viable microorganisms and bacterial components, such as LPSs, originating from subgingival plaque, together with pro-inflammatory cytokines released from inflamed periodontal tissues, including TNF-α, IL-1β, IL-6, IL-8, and CRP, can enter the systemic circulation and contribute to the development of a maternal systemic inflammatory response [145,146]. Pregnancy itself represents a physiologically stressful condition characterized by heightened inflammatory activity, increased gingival inflammation, and progressive insulin resistance [147,148]. Persistent elevation in pro-inflammatory cytokines, particularly IL-1β and TNF-α, has been associated with pancreatic β-cell dysfunction and destruction [149,150,151]. Therefore, chronic maternal periodontal disease may induce a sustained systemic inflammatory response capable of exacerbating insulin resistance. This infection-induced insulin resistance may further amplify the physiological insulin resistance associated with pregnancy, ultimately leading to impaired glucose tolerance and the development of GDM. However, current evidence is largely observational, and further prospective and interventional studies are needed to clarify whether periodontal disease represents an independent risk factor for gestational diabetes mellitus.

Overall, current evidence supports an association between oral microbiota dysbiosis, particularly periodontal disease, and adverse pregnancy outcomes. While differences exist among studies regarding the specific bacterial taxa involved, the most consistent findings relate to enrichment of periodontal pathogens, increased inflammatory burden, and activation of the oral–placental axis as potential mechanisms linking oral dysbiosis to obstetrical complications. However, the available evidence remains heterogeneous, reflecting variability in study populations, periodontal disease definitions, microbiological assessment methods, and control of confounding factors. Moreover, most available evidence originates from observational and epidemiological studies, whereas mechanistic pathways have been investigated primarily in experimental models. Therefore, although the association between oral dysbiosis and adverse pregnancy outcomes is increasingly supported by the literature, direct causal relationships and the magnitude of the clinical impact remain subjects of ongoing debate.

Studies investigating the association between oral microbiota dysbiosis and obstetrical complications are presented in Table 4.

Although numerous studies have reported associations between periodontal disease and adverse pregnancy outcomes, the overall body of evidence remains heterogeneous. Differences in study design, sample size, microbiological assessment techniques, and adjustment for confounding variables may contribute to inconsistencies across studies. Consequently, while biological plausibility is supported by experimental data, further longitudinal and interventional studies are required to clarify the clinical significance of oral microbiota dysbiosis during pregnancy.

### 3.5. An Overview of Maternal Microbiota- Placenta Axis

The human microbiota represents a complex and dynamic ecosystem of microorganisms colonizing multiple anatomical niches, including the oral cavity, gastrointestinal tract, and female reproductive tract. During pregnancy, these microbial communities play a significant role in shaping maternal immune responses, metabolic homeostasis, and placental development. Increasing evidence suggests that the oral, gut, and vaginal microbiota form an interconnected biological axis capable of influencing pregnancy outcomes through microbial translocation, immune modulation, and inflammatory pathways [4,24].

The oral, gut, and vaginal microbiota should not be viewed as isolated microbial ecosystems but rather as components of an integrated maternal microbiota axis. Microbial interactions between these niches may occur through systemic circulation, immune signaling pathways, and microbial metabolites. For example, oral pathogens may reach the gut through saliva ingestion, while intestinal microbial metabolites can influence vaginal immunity and microbial balance. This interconnected microbial network suggests that disturbances in one microbial niche may have cascading effects on others, ultimately influencing the maternal–fetal interface. Consequently, maintaining microbial balance across these ecosystems may represent an important strategy for promoting healthy pregnancy outcomes.

Collectively, alterations in the oral, gut, and vaginal microbiota may influence the maternal systemic environment and the placental microenvironment through complex immune and metabolic pathways. These interactions have been implicated in the development of several obstetric complications, including PTB, preeclampsia, GDM, and intrauterine growth restriction [119]. Understanding the mechanisms linking maternal microbiota to placental function may open new perspectives for preventive strategies, diagnostic biomarkers, and microbiota-targeted therapeutic interventions aimed at improving maternal and fetal health outcomes.

**Figure 4:**
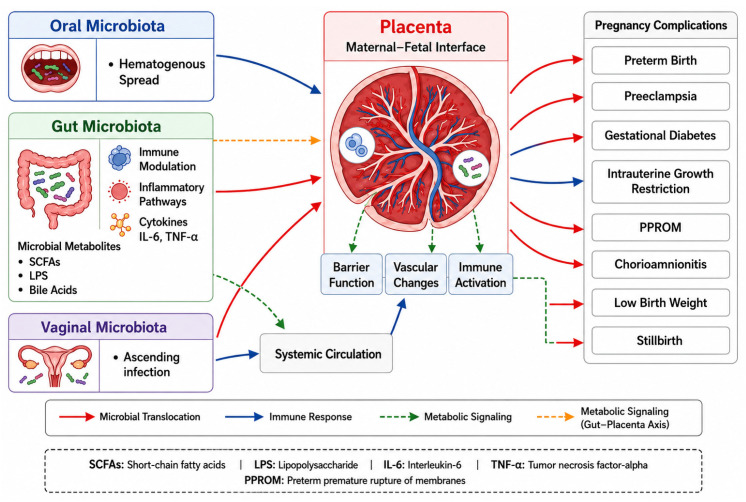
**Maternal microbiota–placenta axis and mechanisms linking maternal microbial communities with adverse pregnancy outcomes.** Oral, gut, and vaginal microbiota can influence placental function through microbial translocation, immune activation, and metabolic signaling. Hematogenous dissemination of oral pathogens, gut microbiota-derived metabolites and cytokines (SCFAs, LPSs, IL-6, TNF-α), as well as ascending infections associated with vaginal microbiota contribute to alterations in placental barrier integrity, vascular remodeling, and immune activation at the maternal–fetal interface. These mechanisms are implicated in the development of pregnancy complications, including preterm birth, preeclampsia, gestational diabetes, intrauterine growth restriction, PPROM, chorioamnionitis, low birth weight, and stillbirth. Red arrows indicate microbial translocation, blue arrows represent immune responses, and green and orange dashed arrows illustrate metabolic signaling pathways.

The Figure 4 highlights the concept of a maternal microbiota–placenta axis, in which microbial imbalance across different body sites may influence placental health and contribute to the development of pregnancy complications.

## 4. Microbiota-Targeted Therapeutic and Preventive Strategies

### 4.1. Clinical Relevance of Maternal Microbiota in Obstetrical Complications

Increasing evidence suggests that maternal microbiota from different body sites, including the vaginal, gut, and oral microbiome, plays an essential role in pregnancy outcomes. Dysbiosis have been associated with several obstetrical complications such as PTB, preeclampsia, GDM, premature rupture of membranes, and intrauterine infections. Understanding the clinical relevance of maternal microbiota may improve early diagnosis, prevention, and management of these complications [75].

The vaginal microbiota is considered one of the most important microbial ecosystems influencing pregnancy outcomes. In healthy pregnancies, the vaginal microbiome is typically dominated by *Lactobacillus* species, which maintain an acidic vaginal environment through lactic acid production and inhibit pathogenic microorganisms. However, vaginal dysbiosis characterized by decreased *Lactobacillus* abundance and increased anaerobic bacteria has been strongly associated with adverse pregnancy outcomes.

Several studies have demonstrated that microbial communities enriched with *Gardnerella vaginalis*, *Fannyhessea vaginae*, *Prevotella species*, and *Sneathia species* are associated with an increased risk of PTB and premature rupture of membranes [75]. A large microbiome study analyzing vaginal samples from more than 1500 pregnant women reported that reduced *Lactobacillus* dominance was strongly associated with spontaneous PTB [20]. These findings suggest that vaginal microbiome profiling may help identify women at increased risk for obstetrical complications.

From a clinical perspective, screening for BV and other forms of vaginal dysbiosis during pregnancy may allow for early identification of high-risk patients. Additionally, emerging research suggests that probiotic therapy targeting *Lactobacillus* species may help restore vaginal microbial balance and potentially reduce the risk of PTB, although further randomized controlled trials are required.

Given the strong association between vaginal microbiota composition and pregnancy outcomes, researchers are increasingly investigating the potential of microbiome-based diagnostic tools and therapeutic strategies.

Studies suggest that vaginal microbiome profiling during early pregnancy may help identify women at risk for PTB or other complications. Additionally, microbiome-modulating therapies such as probiotics, antibiotics targeting BV, and microbial transplantation are currently being explored as potential preventive strategies [28].

However, despite promising findings, clinical evidence remains inconsistent, and further large-scale randomized controlled trials are needed to determine whether microbiome-targeted interventions can effectively reduce the incidence of adverse pregnancy outcomes.

The maternal gut microbiota also plays a crucial role in metabolic and immune regulation during pregnancy. Dysbiosis of intestinal microbial communities has been associated with several obstetrical complications, particularly GDM and preeclampsia.

A study analyzing gut microbiota in women with GDM reported significant differences in microbial composition compared with healthy pregnancies, including increased abundance of pro-inflammatory bacteria and decreased levels of beneficial microbial taxa involved in metabolic regulation [25]. These microbial alterations were associated with insulin resistance and metabolic dysregulation.

Similarly, studies investigating gut microbiota in women with preeclampsia have reported reduced microbial diversity and increased abundance of bacteria associated with systemic inflammation [121]. Gut microbiota dysbiosis may contribute to pregnancy complications through increased intestinal permeability, allowing microbial endotoxins such as LPSs to enter the bloodstream and trigger inflammatory pathways affecting placental function.

Clinically, these findings highlight the potential of microbiome-based biomarkers for identifying women at risk of metabolic pregnancy complications. Dietary interventions and probiotic supplementation targeting gut microbiota are currently being investigated as potential therapeutic strategies to improve maternal metabolic health during pregnancy.

The maternal oral microbiome has also been linked to pregnancy complications, particularly through the association between periodontal disease and adverse pregnancy outcomes. Periodontal pathogens such as *Fusobacterium nucleatum*, *Porphyromonas gingivalis*, and *Prevotella intermedia* have been detected in placental tissues and amniotic fluid in some studies, suggesting that oral bacteria may reach the placenta via hematogenous dissemination [74].

Clinical studies have shown that maternal periodontal disease is associated with increased risk of preterm birth, low birth weight, and preeclampsia. These findings suggest that oral health during pregnancy may influence systemic inflammatory responses and placental function. From a clinical standpoint, improving maternal oral health through periodontal care during pregnancy may represent an important preventive strategy for reducing the risk of obstetrical complications. The growing recognition of the role of maternal microbiota in pregnancy outcomes has led to increasing interest in strategies aimed at modulating microbial communities during gestation. Microbiota-targeted interventions seek to restore microbial balance and improve maternal–fetal health by influencing microbial diversity, metabolic activity, and immune signaling. These strategies include dietary interventions, probiotics, prebiotics and lifestyle modifications, which may alter microbial composition in the maternal gut, vaginal, and oral ecosystems [21].

Research suggests that maternal microbiota modulation during pregnancy may influence metabolic regulation, immune tolerance, placental function, and neonatal microbial colonization. Because maternal microbial communities serve as the primary source of microbial transmission to the infant, modifying maternal microbiota during pregnancy may also have long-term implications for offspring health [162].

### 4.2. Probiotics and Prebiotics

Probiotics are defined as live microorganisms that confer health benefits to the host when administered in adequate amounts. During pregnancy, probiotic supplementation has been investigated as a potential strategy to modulate maternal microbiota and reduce the risk of pregnancy complications.

Several randomized clinical trials have evaluated the effects of probiotic supplementation during pregnancy on maternal metabolic health. Probiotic containing *Lactobacillus* and *Bifidobacterium* species supplementation in pregnant women improved glucose metabolism and reduced markers of systemic inflammation in women with GDM. Moreover, probiotic supplementation may improve insulin sensitivity by restoring microbial balance and reducing intestinal permeability [163].

More recent research has also investigated the influence of probiotics on maternal and neonatal microbiota. In a study examining maternal-to-neonatal microbial transmission, prenatal probiotic supplementation altered the microbial composition of neonatal meconium and increased microbial stability during the early postnatal period [162].

These findings suggest that probiotic use during pregnancy may influence the early establishment of the infant microbiome.

Another observation is that probiotic supplementation during pregnancy is associated with reduced inflammatory biomarkers in breast milk and increased abundance of beneficial bacteria such as *Bifidobacterium* and *Lactobacillus* in the infant gut microbiome [164]. These results highlight the potential role of probiotics in shaping early microbial colonization and immune development.

However, evidence regarding the ability of probiotics to prevent obstetrical complications remains inconsistent. A recent umbrella review evaluating probiotic interventions during pregnancy reported that while probiotics can modulate maternal gut microbiota and improve metabolic parameters, their effects on major obstetrical outcomes such as preeclampsia and preterm birth remain inconclusive [165].

Overall, current evidence suggests that probiotic supplementation during pregnancy may contribute to improved metabolic regulation, immune modulation, and neonatal microbial colonization, although further large randomized controlled trials are needed to confirm their effectiveness in preventing pregnancy complications.

Prebiotics are non-digestible dietary compounds that selectively stimulate the growth and activity of beneficial microorganisms in the gut microbiota. Common prebiotics include galacto-oligosaccharides (GOSs), fructo-oligosaccharides (FOSs), and inulin, which serve as substrates for beneficial bacteria such as *Bifidobacterium* and *Lactobacillus* species.

Recent studies have demonstrated that maternal prebiotic supplementation during pregnancy can significantly modify maternal gut microbiota composition and metabolic activity. For instance, a study investigating maternal supplementation with GOS/FOS (14.2 g per day) reported increased abundance of beneficial microbial taxa and improved microbial metabolic pathways in pregnant women [166]. Importantly, the study also demonstrated that maternal prebiotic intake influenced the developing infant gut microbiome, suggesting that dietary modulation during pregnancy can shape early microbial colonization.

Experimental studies have also suggested that prebiotic supplementation may influence immune regulation during pregnancy. In one study, maternal prebiotic supplementation increased the abundance of Tregs and regulatory B cells in gestational tissues and fetal immune compartments, indicating that prebiotics may promote immune tolerance during pregnancy [167].

In addition to immune effects, prebiotics may influence microbial metabolite production. Fermentation of prebiotic fibers by gut bacteria results in the production of SCFAs, such as acetate, propionate, and butyrate. These metabolites play an important role in maintaining intestinal barrier integrity, regulating immune responses, and supporting metabolic homeostasis during pregnancy [16].

Recent research highlights that maternal microbiota can be influenced by several modifiable factors during pregnancy, including diet, supplementation, and lifestyle factors. For example, nutritional supplementation studies have demonstrated that balanced maternal dietary interventions can alter maternal gut microbiome diversity and metabolic pathways, with potential benefits for maternal immune and metabolic health [168].

Similarly, longitudinal studies examining maternal microbiome dynamics have shown that maternal diet, probiotic supplementation, and environmental exposures may influence both maternal microbial composition and neonatal microbial colonization patterns [162].

Taken together, these findings suggest that microbiota-targeted interventions during pregnancy represent a promising area for preventive strategies aimed at improving maternal and neonatal health. However, further research is required to determine optimal microbial strains, dosages, and intervention timing for achieving clinically meaningful outcomes.

### 4.3. Antibiotic Therapy: Benefits and Limitations in Preventing Infection-Related Obstetrical Complications

Antibiotic therapy is an essential component of obstetrical care for preventing infection-related complications during pregnancy. However, increasing recognition of the impact of antibiotics on maternal and neonatal microbiota has highlighted the importance of antibiotic stewardship, defined as the responsible and evidence-based use of antibiotics to optimize clinical outcomes while minimizing unnecessary exposure, antimicrobial resistance, and disruption of beneficial microbial communities. Infections such as group *B Streptococcus* (GBS) colonization, urinary tract infections, and intrauterine infections associated with premature rupture of membranes represent major causes of maternal and neonatal morbidity. Consequently, targeted antibiotic treatment and prophylaxis are widely used to reduce infection-related obstetrical complications [169].

One of the most successful examples of antibiotic use in obstetrics is intrapartum antibiotic prophylaxis (IAP) for preventing early-onset neonatal GBS infection. Recent studies confirm that IAP significantly reduces the risk of neonatal sepsis caused by *Streptococcus agalactiae*, making it a key preventive intervention in modern obstetrical practice [170].

Antibiotic therapy also plays an important role in the management of premature rupture of membranes. Clinical trials and observational studies demonstrate that antibiotic administration in premature rupture of membranes can prolong the latency period between membrane rupture and delivery, reduce maternal chorioamnionitis, and decrease neonatal infection rates [171]. Recent clinical guidelines recommend combinations such as ampicillin or amoxicillin with macrolides as first-line therapy for infection prevention in premature rupture of membranes cases [172].

Similarly, antibiotic treatment of asymptomatic bacteriuria during pregnancy significantly reduces the risk of maternal pyelonephritis and adverse pregnancy outcomes. Screening and treatment programs for bacteriuria during pregnancy are therefore widely recommended in obstetrical practice [169].

In addition to conventional antibiotic therapy, antiseptic-based approaches have been explored as potential alternatives or adjunctive strategies for the management of bacterial vaginosis. Interest in these interventions has increased because antiseptics may provide broad antimicrobial activity while reducing some concerns associated with repeated antibiotic exposure and the emergence of antimicrobial resistance. Although evidence regarding their use during pregnancy remains limited, topical antiseptic and microbicidal formulations have been proposed as potential options for reducing vaginal pathogen burden and restoring vaginal health. Furthermore, studies evaluating pregnant women’s perceptions of topical microbicides have reported generally favorable attitudes toward these non-antibiotic approaches, suggesting good acceptability for preventive and therapeutic use during pregnancy [173]. Nevertheless, additional clinical studies are required to establish their safety, efficacy, and impact on both vaginal microbiota composition and obstetrical outcomes.

These considerations underscore the importance of adhering to antibiotic stewardship principles during pregnancy. Appropriate antibiotic selection, dose optimization, treatment duration, and avoidance of unnecessary antibiotic exposure are essential for maximizing therapeutic benefits while minimizing adverse effects on maternal and neonatal microbiota.

Despite these benefits, increasing evidence suggests that antibiotic exposure during pregnancy may have unintended consequences due to disruption of maternal and neonatal microbiota. Antibiotics can significantly alter microbial diversity and reduce the abundance of beneficial microorganisms such as *Bifidobacterium* and *Lactobacillus*, which play an important role in immune development and metabolic regulation [174].

Recent studies have shown that prenatal or intrapartum antibiotic exposure can influence early neonatal microbiome development. For example, IAP significantly altered the early development of neonatal oral and gut microbiomes in the first days after birth [175]. Another investigation reported that antibiotic exposure during pregnancy was associated with reduced microbial diversity and altered gut microbiota composition in infants, suggesting long-term effects on microbial colonization [176].

Research published in 2025 also reported that infants exposed to prenatal antibiotics exhibited lower gut microbiota diversity and increased abundance of certain opportunistic bacteria, indicating persistent microbiome alterations during early life [177]. These findings highlight the potential long-term consequences of maternal antibiotic exposure on infant microbial development.

Another concern is the potential link between antibiotic-induced microbiome disruption and immune-related diseases. Studies suggest that alterations in the early infant microbiome may affect immune system development and could increase susceptibility to allergic and inflammatory diseases later in life [178].

### 4.4. Diet and Lifestyle Interventions: Impact of Maternal Nutrition on Gut Microbiota

Maternal diet represents one of the most important modifiable factors influencing the composition and function of the gut microbiota during pregnancy. Nutritional intake shapes microbial diversity, metabolic activity, and the production of microbial metabolites that regulate immune responses and metabolic homeostasis. Increasing evidence suggests that maternal dietary patterns during pregnancy can significantly influence gut microbiota composition, thereby affecting maternal metabolic health, placental function, and fetal development [179].

Pregnancy is associated with physiological changes in the maternal microbiome, including shifts in microbial diversity and abundance across trimesters. In a longitudinal study analyzing fecal microbiota during pregnancy, Koren et al. reported that gut microbiota composition changes significantly during gestation, particularly during the third trimester, with increased abundance of *Proteobacteria* and *Actinobacteria* and reduced microbial diversity. These microbial shifts were associated with metabolic adaptations similar to those observed in metabolic syndrome, including increased insulin resistance and adiposity [3]. Although these changes are considered part of normal pregnancy physiology, maternal diet may further influence these microbial patterns.

Dietary patterns rich in fiber, fruits, vegetables, and whole grains are associated with increased microbial diversity and enrichment of beneficial bacterial taxa such as *Bifidobacterium* and *Lactobacillus* species. These microorganisms ferment dietary fibers into SCFAs, including acetate, propionate, and butyrate, which play important roles in maintaining intestinal barrier integrity, regulating immune responses, and modulating glucose metabolism [180]. SCFAs have also been shown to influence placental function and fetal metabolic programming, highlighting the importance of maternal nutrition in shaping the maternal–fetal metabolic environment [181].

In contrast, diets characterized by high consumption of processed foods, saturated fats, and refined sugars have been associated with reduced microbial diversity and increased abundance of pro-inflammatory microbial taxa. A study examining the effects of maternal dietary patterns on gut microbiota composition demonstrated that Western-style diets were associated with increased levels of *Firmicutes* and *Proteobacteria*, which are linked to systemic inflammation and metabolic disturbances [182]. Such microbial alterations may contribute to pregnancy complications such as GDM and preeclampsia.

Several studies have also investigated the effects of specific dietary interventions on maternal gut microbiota during pregnancy. For example, a recent randomized controlled trial evaluating a Mediterranean-style dietary intervention during pregnancy reported increased microbial diversity and higher abundance of beneficial bacterial taxa in the intervention group compared with controls. The study also observed improvements in maternal metabolic parameters, suggesting that dietary interventions may influence both microbial composition and metabolic health during pregnancy [183].

In addition to diet, lifestyle factors such as physical activity, stress, sleep patterns, and antibiotic exposure can also influence maternal gut microbiota composition. Regular physical activity has been associated with increased microbial diversity and improved metabolic regulation, whereas chronic stress and sleep disturbances may contribute to microbial dysbiosis through alterations in immune and neuroendocrine pathways [184].

Because maternal microbiota represents the primary source of microbial colonization for the newborn, maternal dietary and lifestyle interventions during pregnancy may also influence the early establishment of the neonatal microbiome. Studies have demonstrated that maternal diet during pregnancy can affect microbial composition in neonatal meconium and infant gut microbiota during early life, suggesting that maternal nutrition plays an important role in shaping the early microbial environment of the offspring [185].

Overall, these findings highlight the importance of maternal dietary patterns and lifestyle factors in shaping gut microbiota composition during pregnancy. Targeted nutritional interventions may therefore represent a promising strategy for promoting microbial balance, improving maternal metabolic health, and reducing the risk of pregnancy complications.

**Figure 5:**
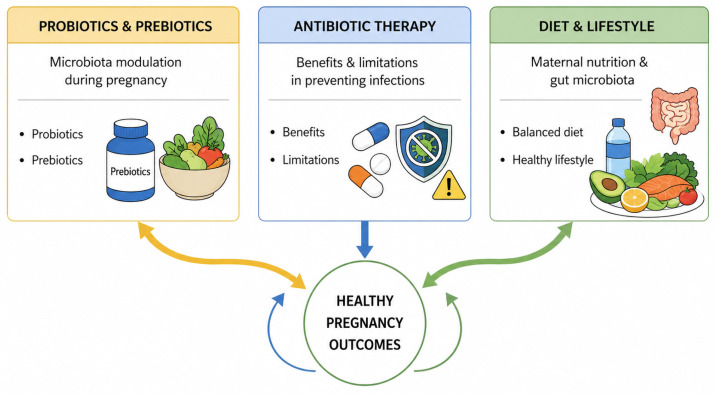
**Microbiota-targeted therapeutic and preventive strategies during pregnancy.** Probiotics and prebiotics support the growth of beneficial microorganisms and help maintain microbial balance. Antibiotic therapy may prevent or treat infection-related complications but must be used cautiously due to potential disruption of microbial communities. Diet and lifestyle interventions, including balanced nutrition rich in fiber and healthy nutrients, influence gut microbiota composition and metabolic regulation. Together, these strategies may contribute to maintaining microbial homeostasis and improving pregnancy outcomes.

Figure 5 presents key therapeutic and preventive approaches targeting the microbiota in pregnancy.

### 4.5. Oral Hygiene and Mechanical Biofilm Control During Pregnancy

Given the growing evidence linking oral dysbiosis and periodontal inflammation to adverse pregnancy outcomes, preventive oral hygiene strategies deserve consideration among microbiota-targeted interventions during pregnancy. Mechanical control of dental biofilm remains the cornerstone of periodontal disease prevention and management, as biofilm accumulation represents the primary etiological factor underlying gingivitis and periodontitis. Pregnancy-associated hormonal and immunological changes increase gingival vascularity, gingival crevicular fluid production, and susceptibility to plaque-induced inflammation, making effective biofilm control particularly important during gestation [186,187].

While conventional toothbrushing remains essential for daily oral hygiene, it has limited effectiveness in disrupting interdental biofilm, where periodontal pathogens frequently accumulate. Consequently, increasing attention has been directed toward interdental cleaning methods, including interdental brushes and dental floss, as adjunctive strategies for controlling biofilm and reducing gingival inflammation [186,187,188].

Recent clinical investigations have suggested that interdental biofilm control may significantly improve periodontal health during pregnancy. In a secondary analysis of a randomized controlled trial, Carrouel et al. reported that calibrated interdental brushing was associated with a significant reduction in gingival bleeding and local inflammatory signs in pregnant women [186]. Similarly, oral prophylaxis strategies targeting interdental biofilm have been shown to reduce the abundance of periodontal pathogens belonging to the red complex, including *Porphyromonas gingivalis*, *Treponema denticola*, and *Tannerella forsythia*, microorganisms frequently associated with periodontal inflammation and adverse pregnancy outcomes [187].

In addition to reducing microbial burden, effective interdental biofilm control may decrease local and systemic inflammatory responses. Periodontal inflammation is characterized by increased production of pro-inflammatory cytokines, including TNF-α, IL-1β, and IL-6, which may enter the systemic circulation and contribute to maternal inflammatory burden. Therefore, reduction in periodontal inflammation through improved oral hygiene practices represents a biologically plausible strategy for mitigating inflammation-associated pathways implicated in obstetrical complications [188,189].

However, despite these promising findings, the current evidence primarily supports the beneficial effects of mechanical biofilm control on periodontal parameters and gingival inflammation. Direct evidence demonstrating that interdental cleaning or other oral hygiene interventions reduce the incidence of adverse pregnancy outcomes, such as preterm birth, preeclampsia, or gestational diabetes mellitus, remains limited. Furthermore, the available studies vary considerably regarding study design, intervention protocols, and clinical endpoints [186,187].

Consequently, mechanical oral biofilm control should currently be regarded as a safe, low-cost, and biologically plausible preventive strategy that may contribute to improved maternal oral health and reduced inflammatory burden during pregnancy. Future longitudinal studies and randomized clinical trials are needed to determine whether improvements in periodontal health achieved through interdental cleaning can translate into measurable benefits for obstetrical outcomes.

## 5. Future Perspectives

Despite the growing evidence linking maternal microbiota to pregnancy outcomes, many questions remain regarding the mechanisms through which microbial communities influence maternal–fetal health. Future research will likely focus on several key areas.

One important direction involves longitudinal cohort studies that follow women throughout pregnancy, allowing researchers to monitor microbial changes across different trimesters and to correlate these changes with pregnancy outcomes. Such studies have shown that the maternal microbiota undergoes dynamic shifts during pregnancy and that these alterations may be associated with complications such as GDM or preterm birth [3].

Another promising research area is the integration of multi-omics approaches, including metagenomics, metabolomics, and transcriptomics. These technologies enable a more comprehensive analysis of microbial composition and function, as well as the metabolic pathways through which microbial communities interact with the host. Recent studies suggest that microbial metabolites, including SCFAs, may influence immune regulation, placental function, and fetal metabolic development [190].

Future studies should focus on the translation of microbiome research into clinical practice. Advances in high-throughput sequencing and multi-omics technologies are expected to improve the identification of microbial biomarkers predictive of obstetrical complications. Integrating microbiome sequencing with metabolomic and immune profiling may enable earlier identification of women at risk for complications such as PTB or GDM [191]. Identifying microbial biomarkers associated with pregnancy complications may allow for earlier risk assessment and the development of targeted preventive strategies. In addition, microbiome-based interventions such as dietary modifications, probiotics, and other microbiota-modulating therapies are being explored as potential approaches to improve pregnancy outcomes [21].

Another promising area of research involves the development of microbiome-targeted interventions. Strategies such as probiotic supplementation, dietary modulation, and microbiota transplantation are being investigated as potential methods for restoring microbial balance during pregnancy. Understanding the physiological dynamics of the vaginal microbiota during pregnancy may therefore provide new opportunities for improving maternal and neonatal outcomes [61]. However, additional clinical trials are required to determine the safety and effectiveness of these approaches in pregnant populations [192].

Advances in microbiome research have opened new perspectives for the development of microbiome-based therapeutic strategies aimed at improving maternal and fetal health during pregnancy. Increasing evidence suggests that maternal microbial communities play an important role in regulating immune responses, metabolic homeostasis, and placental function. Consequently, targeted modulation of maternal microbiota may represent a promising strategy for preventing pregnancy complications such as PTB, GDM, and preeclampsia [45].

One emerging approach involves the application of personalized medicine strategies based on individual microbiome profiles. Recent studies have demonstrated that microbial composition varies significantly among individuals, influenced by factors such as diet, genetics, environment, and antibiotic exposure. Integrating microbiome sequencing with clinical and metabolic data may allow clinicians to identify microbial biomarkers associated with increased risk of pregnancy complications and to develop individualized preventive strategies [193]. For example, microbial profiling early in pregnancy has been proposed as a potential tool for predicting metabolic disorders such as GDM and guiding targeted dietary or probiotic interventions.

Another promising area of research involves microbiome modulation as a preventive strategy. Interventions aimed at restoring microbial balance—including probiotics, prebiotics, synbiotics, and dietary modifications—are currently being investigated for their potential to improve pregnancy outcomes. These strategies may promote the growth of beneficial microorganisms such as *Lactobacillus* and *Bifidobacterium* species, which are involved in immune regulation and metabolic stability [164]. In addition, emerging therapeutic approaches such as microbiota transplantation and microbial metabolite-based therapies are being explored as potential methods for correcting microbial dysbiosis associated with pregnancy complications [194].

Future obstetrical care may incorporate personalized microbiome analysis to guide prevention and treatment strategies. Individual variations in microbial composition may influence how pregnant women respond to dietary interventions, probiotics, or pharmacological therapies. Personalized microbiome-based approaches may therefore improve pregnancy outcomes and reduce complications.

Another important research direction involves the study of interactions between different maternal microbiomes, including the gut–vagina–oral microbiome axis. Understanding how these microbial ecosystems interact during pregnancy may provide insights into the mechanisms linking maternal microbial health with fetal development and pregnancy complications [45].

However, further research using contamination-controlled sequencing and standardized methodologies is necessary to clarify whether a true placental microbiome exists and to determine the precise role of microbial components in pregnancy physiology.

Although microbiome-based therapies represent a promising field, further research is required to determine the optimal microbial targets, intervention timing, and long-term safety of these approaches during pregnancy. Future studies integrating microbiome sequencing, metabolomics, and clinical trials will be essential for translating microbiome research into effective preventive and therapeutic strategies in obstetric care.

## 6. Conclusions

Recent advances in microbiome research have significantly improved our understanding of the role of maternal microbial communities in pregnancy. The vaginal, gut, and oral microbiota contribute to maintaining physiological homeostasis through their involvement in immune regulation, metabolic balance, and protection against pathogenic microorganisms. Under normal conditions, these microbial ecosystems support maternal–fetal health by maintaining mucosal stability and preventing ascending infections.

However, disturbances in microbial composition, known as microbial dysbiosis, have been increasingly associated with several obstetrical complications, including PTB, premature rupture of membranes, GDM, preeclampsia, and intrauterine infections. Altered microbial diversity, the reduction in protective bacteria such as *Lactobacillus*, and the proliferation of pathogenic microorganisms may contribute to inflammation, immune dysregulation, and metabolic disturbances that negatively affect pregnancy outcomes.

Current evidence also highlights the important role of maternal microbiota in early neonatal microbial colonization and immune development. Maternal microbial communities therefore represent a key link between maternal health and long-term health outcomes in the offspring.

Although the exact mechanisms linking microbiota dysbiosis with obstetrical complications are still being investigated, emerging research suggests that microbiome-based approaches may offer new opportunities for prevention and treatment. Strategies such as microbiota-targeted therapies, probiotic and prebiotic supplementation, dietary interventions, and personalized microbiome-based medicine may contribute to improving maternal and neonatal health in the future.

Further research, particularly longitudinal cohort studies and multi-omics approaches, will be essential for clarifying the complex interactions between maternal microbiota and pregnancy physiology. A better understanding of these mechanisms may ultimately allow for the integration of microbiome research into clinical practice, leading to innovative strategies for preventing obstetrical complications and improving pregnancy outcomes.

## Figures and Tables

**Table 1:** Dominant bacterial communities and trimester-specific physiological changes in the maternal bacteriome during healthy pregnancy. Information summarized from studies by Ravel et al. [14], Koren et al. [3], and Dewhirst et al. [50].

Body Site	Dominant Bacteria Taxa	First Trimester	Second Trimester	Third Trimester
Vaginal	*Lactobacillus* spp.	*Lactobacillus* dominance established	Reduced diversity and increased stability	Maximal *Lactobacillus* dominance
Gut	*Firmicutes*, *Bacteroidetes*	Similar to non-pregnant state	Changes in SCFA-producing taxa	Increased *Proteobacteria* and *Actinobacteria*
Oral	*Firmicutes*, *Proteobacteria*, *Actinobacteria*	Relatively stable	Increased *Prevotella* abundance	Increased periodontal-associated bacteria

**Table 2:** **Studies investigating the association between vaginal microbiota dysbiosis and obstetrical complications**.

Author	Study Design	Outcome Assessed	Key Findings	Clinical Relevance
Fettweis et al. (2019) [20]	16S rRNA vaginal microbiome sequencing	Spontaneous preterm birth	Reduced *Lactobacillus* dominance and distinct microbial community profiles in PTB cases	Vaginal microbiota composition may serve as a biomarker of PTB risk
Stinson et al. (2019) [70]	Molecular sequencing of amniotic fluid	Intrauterine inflammation, fetal exposure to microbes	Dysbiosis associated with altered metabolic pathways and increased inflammatory cytokines	Microbiome–metabolome interactions may contribute to PTB pathogenesis
Shen et al. (2025) [69]	Integrated microbiome and metabolomics analysis	Preterm birth	Dysbiosis associated with altered metabolic pathways and elevated inflammatory cytokines	Microbiome–metabolome interactions may contribute to PTB pathogenesis
McKee et al. (2025) [68]	Longitudinal cohort study	Spontaneous preterm birth	Early reduction in *Lactobacillus* dominance predicted PTB	Early microbial alterations may identify women at increased PTB risk
Nam et al. (2025) [71]	Metagenomic sequencing	Preterm premature rupture of membranes	Increased microbial diversity and enrichment of BV-associated bacteria	Vaginal dysbiosis may contribute to membrane instability
Tang et al. (2024) [72]	Vaginal microbiome biomarker study	Preterm premature rupture of membranes	Identification of microbial signatures associated with membrane weakening	Microbial biomarkers may help predict PPROM risk
Cheng et al. (2025) [59]	Mechanistic microbiome and immune signaling study	Preterm birth	BV-associated bacteria activated inflammatory cytokine pathways	Inflammatory activation may contribute to PTB pathophysiology
Deng et al. (2025) [73]	Microbiome and immune biomarker study	Intrauterine inflammation and adverse obstetrical outcomes	Dysbiosis associated with increased inflammatory biomarkers	Microbial imbalance may promote pregnancy-related inflammation
Xie et al. (2025) [45]	Multi-cohort pregnancy microbiome study	Adverse obstetrical outcomes (including preterm birth)	Dysbiotic vaginal microbiota associated with inflammatory pathways	Vaginal microbiota may influence pregnancy outcomes through immune modulation
Crusell et al. (2018) [25]	Gut microbiome cohort study in pregnant women	Gestational diabetes mellitus	Altered gut microbiota composition in women with gestational diabetes compared to healthy pregnancies.	Gut microbial dysbiosis may contribute to metabolic disturbances in gestational diabetes.

**Table 3:** **Gut microbiota dysbiosis in obstetrical complications**.

Reference	Study Design	Outcome Assessed	Key Findings	Clinical Relevance
Crusell et al. (2018) [25]	16S rRNA sequencing of fecal samples	Gestational diabetes mellitus	Altered gut microbiota composition, reduced diversity, and enrichment of pro-inflammatory taxa	Gut dysbiosis may contribute to metabolic and inflammatory mechanisms involved in GDM
Koren et al. (2012) [3]	Longitudinal metagenomic analysis during pregnancy	Metabolic adaptations and insulin resistance	Increased Proteobacteria and Actinobacteria during late pregnancy	Physiological microbiota changes may influence metabolic adaptation to pregnancy
Wang et al. (2020) [117]	Gut microbiota sequencing	Preeclampsia	Reduced microbial diversity and increased inflammatory taxa	Gut microbial imbalance may contribute to preeclampsia-associated inflammation
Xie et al. (2025) [45]	Multi-cohort pregnancy microbiome analysis	Multiple pregnancy complications	Dysbiosis associated with inflammatory and metabolic alterations	Gut microbiota alterations may represent a common pathway across pregnancy complications
Shen et al. (2025) [69]	Integrated microbiome and metabolomics analysis	Preterm birth	Altered microbial metabolites and activation of inflammatory pathways	Microbiome–metabolome interactions may contribute to PTB pathogenesis
Ferrocino et al. (2018) [27]	16S rRNA sequencing of gut microbiota	Gestational diabetes mellitus	Distinct microbial signatures associated with glucose intolerance	Specific microbial profiles may contribute to metabolic dysregulation in GDM
Barnea et al. (2026) [118]	Microbiome and metabolic profiling	Gestational diabetes mellitus	Microbiota alterations associated with insulin resistance and metabolic inflammation	Gut microbiome–metabolism interactions may influence GDM development
Giugliano et al. (2025) [112]	Experimental animal study	Placental dysfunction and impaired fetal development	Induced dysbiosis resulted in placental abnormalities and altered fetal growth	Gut microbiota imbalance may affect placental function and fetal development
Du et al. (2025) [105]	Multi-omics microbiome study	Multiple pregnancy complications	Dysbiosis associated with systemic inflammation and metabolic disturbances	Integrated microbiome alterations may contribute to pregnancy complications
Crusell et al. (2018) [25]	Longitudinal gut microbiome cohort study	Gestational diabetes mellitus	Altered microbial composition and increased inflammatory markers in women with GDM	Gut microbiota composition may serve as an indicator of GDM risk
Gomez-Arango et al. (2017) [119]	Gut microbiome sequencing in obese pregnant women	Gestational diabetes and metabolic dysregulation	Reduced beneficial bacteria and increased inflammatory taxa	Maternal gut microbiota may influence metabolic complications during pregnancy
Zheng et al. (2015) [120]	Gut microbiome and metabolomic profiling	Preeclampsia	Altered microbial metabolites and increased inflammatory bacterial taxa	Gut microbiota may contribute to hypertensive disorders through metabolic and inflammatory pathways
Lv et al. (2019) [121]	16S rRNA gut microbiome sequencing	Preeclampsia	Reduced microbial diversity and increased opportunistic pathogens	Gut dysbiosis may contribute to immune and inflammatory mechanisms in preeclampsia
Kuang et al. (2017) [122]	Gut microbiome sequencing and metabolic analysis	Gestational diabetes mellitus	Altered microbial composition associated with impaired glucose metabolism	Maternal gut microbiota may influence glucose homeostasis during pregnancy

**Table 4:** **Studies on maternal oral microbiota dysbiosis in obstetrical disorders**.

Reference	Study Design	Outcome Assessed	Key Findings	Clinical Relevance
Vidmar Šimic et al. (2023) [139]	16S rRNA sequencing of the oral microbiome	Preterm birth	Increased abundance of *Veillonella*, *Prevotella*, and *Capnocytophaga*	Specific oral bacterial taxa were associated with increased PTB risk
García-Rios et al. (2026) [142]	Cohort study with oral microbiome sequencing	Preterm birth	Increased abundance of Firmicutes and Bacteroidetes in PTB cases	Oral microbial signatures differed between preterm and term pregnancies
Liu et al. (2024) [143]	Case–control salivary microbiome study	Low birth weight, preterm birth	Increased abundance of *Clostridia*, *Leptotrichia buccalis*, and *Gemella sanguinis*	Oral microbiota instability was associated with adverse birth outcomes
Benslimane et al. (2024) [76]	Metabarcoding analysis of oral microbiota	Multiple pregnancy complications	Altered oral microbial diversity during pregnancy	Oral microbiota changes may influence maternal–fetal health
Winckler et al. (2024) [152]	Periodontal treatment intervention study	Preterm birth	Reduction in inflammatory markers following periodontal therapy	Periodontal treatment may improve pregnancy-related inflammatory status
Gilani et al. (2024) [153]	Epidemiological analysis	Preterm birth	Periodontal pathogens were associated with systemic inflammation	Periodontitis may contribute to adverse pregnancy outcomes
Tsikouras et al. (2024) [154]	Clinical observational study	Preeclampsia, preterm birth	Periodontal pathogens were associated with increased inflammatory burden	Oral infections may contribute to obstetrical complications
Castaño-Suárez et al. (2024) [155]	Meta-epidemiological analysis	Preterm birth	Periodontitis was consistently associated with increased PTB risk	Epidemiological evidence supports an association between periodontitis and PTB
Adebayo et al. (2024) [156]	Epidemiological review of hospital cohorts	Low birth weight, preterm birth	Maternal periodontitis was associated with adverse pregnancy outcomes	Maternal oral disease may represent an obstetrical risk factor
Ohmichi-Tomiwa et al. (2025) [157]	Exploratory oral microbiome study	Multiple pregnancy complications	Altered oral microbiota composition during pregnancy	Oral dysbiosis may be associated with preeclampsia and PTB
Liu et al. (2025) [158]	Microbiome study in women with pregnancy loss	Pregnancy loss	Reduced microbial diversity and altered metabolic pathways	Oral dysbiosis may be associated with increased pregnancy loss risk
Dodson et al. (2025) [140]	Oral microbiome review	Preterm birth, placental infection	Oral bacteria were identified in intrauterine tissues	Hematogenous dissemination may link oral infection and placental inflammation
Zhao et al. (2025) [159]	Bibliometric and clinical analysis	Multiple pregnancy complications	Increasing evidence links oral dysbiosis with adverse obstetrical outcomes	Oral microbiota is increasingly recognized as a relevant factor in pregnancy health
Han et al. (2010) [74]	Clinical and experimental microbiological study	Stillbirth, intrauterine infection	*Fusobacterium nucleatum* was detected in placental tissues	Oral bacteria may reach the placenta through hematogenous spread
Ide & Papapanou (2013) [34]	Systematic review and epidemiological analysis	Preterm birth, low birth weight	Maternal periodontitis was associated with adverse pregnancy outcomes	Periodontal disease may increase the risk of PTB and fetal growth restriction
Offenbacher et al. (2006) [160]	Prospective cohort study	Preterm birth	Increased periodontal pathogens and inflammatory mediators in PTB cases	Periodontal inflammation may contribute to PTB-associated inflammatory pathways
Fardini et al. (2010) [161]	Experimental microbial transmission study	Placental infection, preterm birth	Oral bacteria were detected in placental tissues following systemic dissemination	Experimental evidence supports the oral–placental transmission pathway

## Data Availability

No new data were created or analyzed in this study. Data sharing is not applicable to this article.

## Abbreviations

AhR	aryl hydrocarbon receptor
BV	bacterial vaginosis
CRP	C-reactive protein
DAMP	damage-associated molecular patterns
FGR	fetal growth restriction
FOSs	fructo-oligosaccharides
GBS	group *B Streptococcus*
GDM	gestational diabetes mellitus
GLP-1	glucagon-like peptide-1
GOSs	galacto-oligosaccharides
GPR41/GPR43	G-protein-coupled receptors 41/G-protein-coupled receptors 43
HDAC	histone deacetylase
IAP	intrapartum antibiotic prophylaxis
IgA	immunoglobulin A
IL-1β/IL-6/IL-8	interleukin-1β/interleukin-6/interleukine-8
LPSs	lipopolysaccharides
MyD88	Myeloid Differentiation Primary Response 88
MMPs	matrix metalloproteinases
NF-κB	nuclear factor kappa-light-chain-enhancer of activated B cells
Nrf2	nuclear factor erythroid 2-related factor 2
PAMPs	pathogen-associated molecular patterns
PGE2	prostaglandin E2
PPROM	preterm premature rupture of membranes
PTB	preterm birth
PTL	preterm labor
PYY	peptide YY
SCFAs	short-chain fatty acids
TLRs	toll-like receptors
TNF-α	tumor necrosis factor-α
Tregs	regulatory T cells
TRIF	TIR-domain-containing adapter-inducing interferon-β
